# Predictive modeling to uncover Parkinson’s disease characteristics that delay diagnosis

**DOI:** 10.1038/s41531-025-00923-2

**Published:** 2025-04-02

**Authors:** Tom Hähnel, Tamara Raschka, Jochen Klucken, Enrico Glaab, Jean-Christophe Corvol, Björn H. Falkenburger, Holger Fröhlich

**Affiliations:** 1 Department of Bioinformatics, Fraunhofer Institute for Algorithms and Scientific Computing (SCAI), Sankt Augustin, Germany; 2 Department of Neurology, Medical Faculty and University Hospital Carl Gustav Carus, TUD Dresden University of Technology, Dresden, Germany; 3 Bonn-Aachen International Center for IT, University of Bonn, Bonn, Germany; 4 Biomedical Data Science, Luxembourg Centre for Systems Biomedicine (LCSB), University of Luxembourg, Esch-sur-Alzette, Luxembourg; 5 Luxembourg Institute of Health (LIH), Strassen, Luxembourg; 6 Centre Hospitalier de Luxembourg (CHL), Esch-sur-Alzette, Luxembourg; 7 Sorbonne Université, Paris Brain Institute – ICM, Inserm, CNRS, Assistance Publique Hôpitaux de Paris, Pitié-Salpêtrière Hospital, Department of Neurology, Paris, France; 8 German Center for Neurodegenerative Diseases (DZNE), Dresden, Germany

**Keywords:** Parkinson's disease, Outcomes research

## Abstract

PD patients present with diverse symptoms, complicating timely diagnosis. We analyzed 1124 PD trajectories using a novel model-based approach to estimate whether diagnosis was early or late compared to cohort averages. Higher age, specific non-motor symptoms, and fast disease progression were linked to later diagnosis, while gait impairment led to earlier diagnosis. Our findings are in line with a biological definition of PD that extends beyond classical motor symptoms.

## Introduction

Numerous ongoing clinical trials investigate potentially disease-modifying treatments for Parkinson’s disease (PD), aiming to stop or significantly slow neurodegeneration^1^. As timely treatments will be required to maximize therapeutic benefits, early diagnosis in PD becomes increasingly important. Therefore, identification and understanding of factors associated with delayed diagnosis is crucial. Previous studies defined diagnostic delay as the time between self-recognition of first motor symptom and PD diagnosis and yielded inconsistent findings^2–7^.

Our study aimed to systematically identify demographic and clinical factors linked to delayed PD diagnosis by using longitudinal data from large cohorts. We employed a novel model-based approach to objectively estimate individual diagnostic delay, thereby overcoming several limitations of previous approaches.

Longitudinal data of 1124 people with Parkinson’s disease (PwPD) from the three cohorts PPMI, ICEBERG, and LuxPARK was included into our analysis (Table 1). Mean *patient-reported time to diagnosis*, i.e. the time span between self-recognition of first motor symptom and PD diagnosis, was 0.9 years for PPMI, 1.1 years for ICEBERG and 1.0 years for LuxPARK (Fig. S1), which is in line with previous publications^8^. Using this traditional approach and correlating the *patient-reported time to diagnosis* with demographic factors and clinical symptoms at baseline visit revealed no significant relationships (Table S1/S2, Fig. S2).

**Table 1:** Baseline demographic and clinical characteristics of PPMI, ICEBERG and LuxPARK cohorts

	PPMI	ICEBERG	LuxPARK	PPMI vs ICEBERG *p* values	PPMI vs LuxPARK *p* values	ICEBERG vs LuxPARK *p* values
**Number of PwPD**	409	154	561	··	··	··
**Age, years**	63.0 [55.2-69.3]	63.7 [57.1-69.4]	67.8 [59.4-73.1]	1.0	*<0.0001*	*<0.0001*
**Sex**	··	··	··	0.71	1.0	0.49
Male	66.7% [273]	62.3% [96]	67.6% [379]	··	··	··
Female	33.3% [136]	37.7% [58]	32.4% [182]	··	··	··
**Hoehn & Yahr**	··	··	··	*<0.0001*	*<0.0001*	*0.0076*
H&Y I	43.8% [179]	2.6% [4]	18.7% [105]	··	··	··
H&Y II	56.2% [230]	93.5% [144]	67.0% [376]	··	··	··
H&Y III	0	3.9% [6]	9.1% [51]	··	··	··
H&Y IV	0	0	3.7% [21]	··	··	··
H&Y V	0	0	1.4% [8]	··	··	··
**Disease duration, years**	0.3 [0.2-0.6]	1.2 [0.6-2.3]	2.9 [0.9-6.5]	*<0.0001*	*<0.0001*	*<0.0001*
**Number of visits**	14 [12–16]	5 [4-5]	4 [3–6]	*<0.0001*	*<0.0001*	0.13
**Follow up, years**	7.0 [5.0-7.0]	4.1 [3.0-4.4]	4.0 [2.4-5.0]	*<0.0001*	*<0.0001*	1.0
**UPDRS I**	5 [3–7]	9 [6–12]	9 [5–13]	*<0.0001*	*<0.0001*	1.0
**UPDRS II**	5 [3–8]	8 [5–10]	10 [5–15]	*<0.0001*	*<0.0001*	*0.0026*
**UPDRS III**	20 [14–26]	29 [24–35]	32 [22–44]	*<0.0001*	*<0.0001*	*0.047*
**UPDRS IV**	..	0 [0-0]	0 [0-1]	··	··	*<0.0001*
**PIGD**	0.2 [0.0-0.4]	0.2 [0.0-0.4]	0.4 [0.2-1.0]	0.26	*<0.0001*	*<0.0001*
**MoCA**	28 [26–29]	28 [26–29]	25 [23–28]	0.11	*<0.0001*	*<0.0001*
**SCOPA**	8 [6–12]	11 [7–17]	14 [9–20]	*0.00035*	*<0.0001*	*0.0015*

For the variables sex and H&Y, both relative and absolute frequencies are shown. All other characteristics are reported using medians and the first and third quartiles. UPDRS IV was not assessed at baseline for the PPMI cohort. Corresponding *p* values were corrected for multiple testing. Significant *p* values are emphasized in italic.

*H&Y* Hoehn & Yahr, *MoCA* Montreal Cognitive Assessment, *PIGD* Postural Instability and Gait Dysfunction score, *SCOPA* Scales for Outcomes in Parkinson’s Disease-Autonomic Dysfunction, *UPDRS* Unified Parkinson’s Disease Rating Scale.

**Fig. 1:**
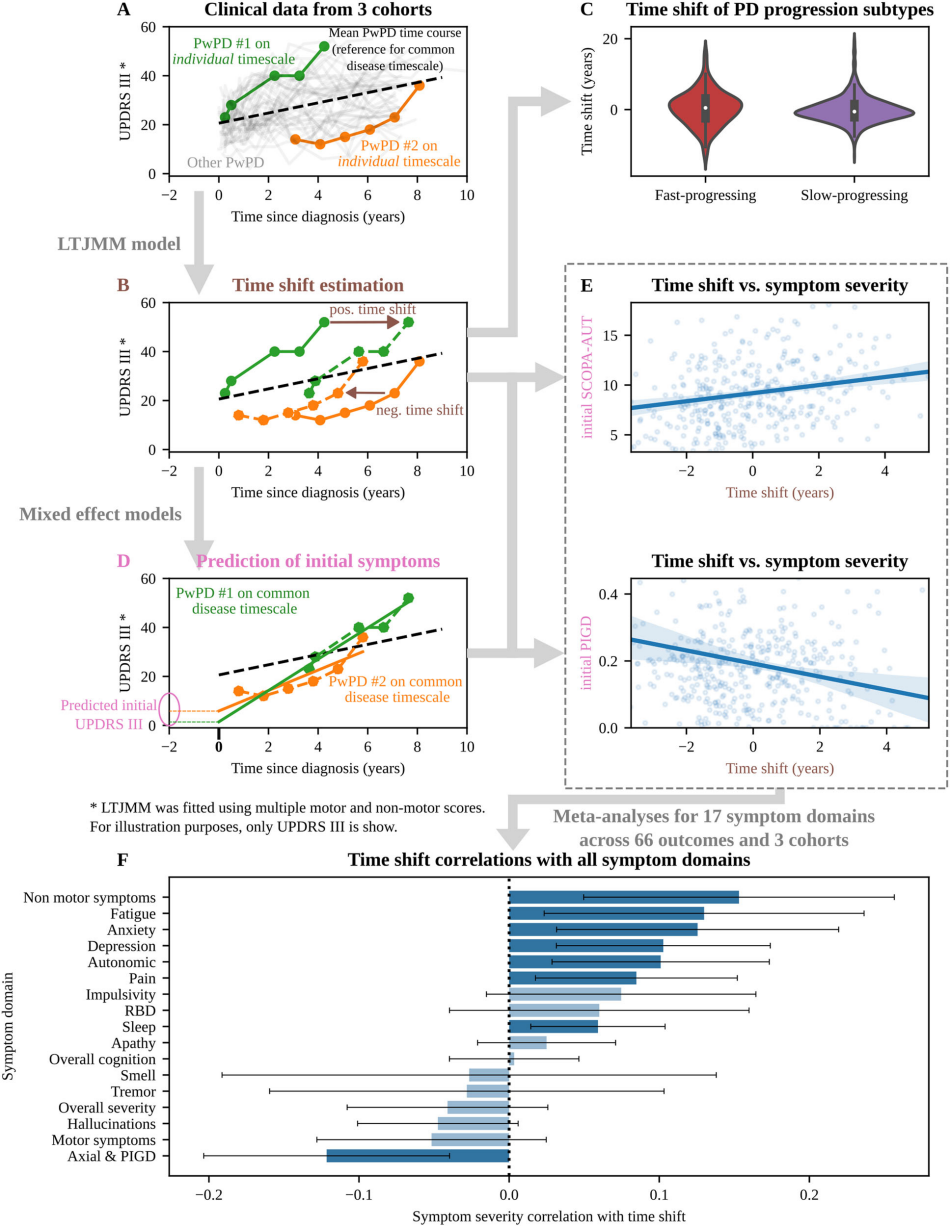
Modeling approach to analyze correlations between initial symptoms and time shifts. **A** We analyzed motor and non-motor outcomes of PwPD from PPMI, ICEBERG and LuxPARK. To illustrate the methodology, we show only UPDRS III trajectories of two PwPD highlighted in orange and green, alongside 50 trajectories from other PwPD in gray. **B** Using a latent time joint mixed-effects model (LTJMM), we calculated how much the trajectory of each PwPD is shifted from the trajectory of the cohort average (dashed black line), i.e. how much earlier or later a diagnosis was made relative to the cohorts average PD trajectory that represents the common disease timescale. For instance, PwPD #1 (green) needs to be shifted right for alignment on the common disease timescale as this PwPD was diagnosed at later disease stage with already more severe symptoms than the average PwPD. PwPD #2 (orange solid line) was diagnosed earlier compared to the average PwPD and needs to be shifted left for alignment on the common disease timescale. Thereby, model-derived time shifts are calculated from multiple outcome trajectories. **C** Violin plots for time shift distributions of the fast-progressing and slow-progressing PD subtype identified in a previous publication. **D** After aligning PwPD on the common disease timescale, progression of all outcomes, including outcomes not used for fitting LTJMM, was modeled using individual mixed-effect models (solid lines). Outcomes were predicted for time = 0 on the common disease timescale to estimate the initial manifestation of each PwPD at a comparable time, i.e. time of typical diagnosis. **E** Subsequently, time shifts (**B**) were correlated with initial symptoms (**D**). Two representative examples are given in the figure. **F** Correlations of all clinical outcomes were aggregated across cohorts into 17 symptom domains. The 95% confidence intervals are shown and were corrected for multiple testing. Significant correlations are indicated in dark blue. Positive correlation coefficients indicate that an increased symptom severity is associated with a later-than-average PD diagnosis (positive time shift). LTJMM latent time joint mixed-effects model, PIGD postural instability and gait disturbance, RBD REM behavior sleep disorder, UPDRS Unified Parkinson’s Disease Rating Scale.

Thus, we next used a model-based approach to objectively estimate diagnostic delay and initial clinical manifestations. A latent time joint mixed-effects model (LTJMM) was fitted on trajectories of motor and non-motor scores of individual PwPD in the three cohorts (Fig. 1A). This model estimates how much individual clinical courses differ from the average PD time course in the cohorts, thereby still accounting for several covariates and inter-individual heterogeneity in disease progression. Since the individual time of diagnosis is known in all PwPD, this modeling approach allows to calculate a time shift for each PwPD indicating whether PwPD were diagnosed earlier or later than average. This is achieved by aligning PwPD on a *common disease timescale* (Figs. 1B and S3/4). We will refer to this model-based definition of diagnostic delay using the terms *earlier-than-average diagnosis* and *later-than-average diagnosis*.

First, we assessed a potential association of later-than-average diagnosis with disease progression, assuming that diagnostic delay *may* in principle be caused by a later medical contact due to a slower disease progression. We analyzed this relationship based on a fast-progressing and slow-progressing PD subtype that we recently identified in the same datasets^9^. Our analysis indicates, however, that the fast-progressing PD subtype is associated with a later-than-average PD diagnosis (*d* = 0.22, *P* = 0.039, Figs. 1C and S5), which is inconsistent with the explanation noted above.

In order to understand this observation, we subsequently explored which demographic attributes and clinical characteristics were associated with early and late PD diagnosis. While the relationship between age and diagnostic delay in PD was previously debated^2–7^, we observed a strong correlation of later-than-average PD diagnosis with higher predicted age at diagnosis, i.e., the patient’s age at the time when PD would be typically diagnosed on the *common disease timescale*. This finding was consistent across all three cohorts and for the pooled analysis (*ρ* = 0.56, *P* < 0.0001, Fig. S6). A plausible explanation of this phenomenon could be that aged PwPD initially attribute their early symptoms to normal aging processes, leading to a delay in seeking medical consultation^10^. Consistent with past literature, we found no impact of predominant PD side or sex on diagnostic delay (Table S3)^2,3,5,6^.

Two reports from Mexico suggested that a positive family history could be associated with higher diagnostic delay, possibly due to cultural factors and the absence of disease-modifying treatments deterring PwPD from seeking medical consultation^5,6^. In contrast, our data collected from many different countries did not find any association between family history and diagnostic delay, thereby indicating potential regional variations in this aspect (Table S3).

Subsequently, we used statistical models to systematically estimate the initial clinical manifestations of individual PwPD, i.e., the symptoms exhibited at the time when PD would be typically diagnosed on the *common disease timescale* (Fig. 1D), and correlated these initial symptoms with the time shifts calculated before (Fig. 1E).

Fatigue, anxiety, depression, autonomic symptoms, pain and sleep disorders were associated with a later-than-average diagnosis of PD (Fig. 1F, Table S4). The non-specific nature of these non-motor symptoms may present a challenge for PwPD to accurately attribute their complaints to the appropriate medical specialty, potentially contributing to delay in the diagnostic process. For example, depressive symptoms may initially lead to psychiatric consultation. Other symptoms, such as constipation, are common in elderly people and may not always lead to consideration of PD as a potential cause. This highlights the need for increased education and awareness among patients and primary care providers, particular if disease-modifying treatments become available. Several of these symptoms had been associated with the fast-progressing subtype, which may explain the correlation of higher diagnostic delay with this subtype^9^.

Accordingly, axial and PIGD (postural instability and gait disturbance) symptoms, which are characteristic of PD, were linked with an earlier-than-average diagnosis (Fig. 1F, Table S4). Noteworthy, no association was identified between tremor and an earlier diagnosis. We hypothesize, that tremor may be perceived by PwPD as a normal age-related condition, thereby potentially delaying medical consultation, despite tremor being a typical and diagnosis-leading symptom for physicians. Contrarily, PIGD symptoms may impact daily living, particularly by their potential to cause falls, which may prompt more urgent medical consultations. Accordingly, on average PwPD of the PIGD subtype were diagnosed earlier as PwPD of the tremor dominant (TD) subtype (Table S3, Fig. S7). While some studies linked higher UPDRS III scores to longer diagnostic delay^4^, we argue that this reflected a reverse causation, where delayed diagnosis resulted in more severe motor symptoms at study inclusion.

Given that symptoms exhibited by female PwPD frequently diverge from those observed in male PwPD^11^, we also investigated sex-specific differences in the relationship between initial symptoms and diagnostic delay. In male PwPD, pain and sleep disturbances did not affect the time to PD diagnosis. In female PwPD, however, impulsivity was linked to later-than-average diagnosis (Table S4).

Age-specific differences were analyzed based on a median split at age 62.2 years. Associations for younger PwPD were mostly similar to the overall findings, except that axial and PIGD symptoms were not correlated with model-derived time shifts. In older PwPD, fewer non-motor symptoms were linked to later-than-average diagnosis, with only increased anxiety being associated with later-than-average diagnosis, whereas pronounced cognitive impairment, motor symptoms, axial and PIGD symptoms, and overall disease severity were linked to earlier-than-average PD diagnosis (Table S4). Potentially, younger individuals may be better able to compensate for axial and PIGD symptoms and delay seeking medical attention. Conversely, older PwPD may experience greater functional impairment from these symptoms, leading to earlier medical consultation. An additional analysis using the official MDS definitions^12^ of early onset PD (EOPD, <50 years) and late onset PD (LOPD, >60 years) subgroups is presented in the supplement (Table S4).

Overall, the impact of non-motor symptoms on diagnostic delay was more pronounced in female and younger PwPD. This might be reflective of the lower PD incidence in these demographic groups and the higher frequency of non-motor symptoms in female PwPD^11^. Consequently, when non-motor symptoms predominate, PwPD and physicians may initially consider other conditions more likely, thereby prolonging the diagnostic process.

In contrast to previous studies^10,13^, our modeling approach does not depend on the patient’s self-perception and memory regarding the time of disease onset and initial complaints. Furthermore, our modeling approach allowed us to use data from structured clinical assessments and questionnaires to provide the first granular and systematic assessment of factors associated with diagnostic delay. Our results were consistent across three cohorts, as shown by the forest plots in the supplement, demonstrating the generalizability of our findings.

Despite these advantages, there are limitations of our modeling approach. The applied models assume an approximately linear progression in outcomes. However, this simplification may not fully acknowledge disease complexity^14^. Despite this simplification, LTJMM has been successfully applied in modeling disease progression in PD^9,15^ and other neurodegenerative disorders^16^. Furthermore, the LTJMM model accounts for inter-individual heterogeneity in disease progression at several levels and corrects for important covariates while aligning PwPD on a *common disease timescale*, as detailed in the methods section.

While our model proficiently estimates diagnostic delay in individuals with PD relative to an average PD disease trajectory, it cannot provide any reasons. It remains open for further research to collect additional data (e.g., from interviews) to assess whether diagnostic delay was due to delayed self-recognition of symptoms or a prolonged diagnostic process. Furthermore, it is tempting to speculate at which timepoint these patients fulfilled current diagnostic criteria for PD^17^. Indeed, neurodegeneration and also non-motor symptoms often precede PD motor symptoms by several years^18–20^. Our findings are therefore consistent with concepts that consider a diagnosis of PD that is based on biological parameters rather than classical motor symptoms^21,22^. Such redefinitions will become increasingly important as new disease-modifying treatments become available, aiming to stop neurodegeneration at its earliest stages. Emerging biomarkers like alpha-synuclein aggregation assays or new digital biomarkers obtained from wearables or digital assessments hold promise for facilitating earlier and more accurate PD diagnosis^23–26^. Digital gait and voice analysis, in particular, could enable large-scale screening to identify individuals at risk for developing PD or those in the prodromal phase. In these high-risk individuals, confirmatory alpha-synuclein aggregation assays could then be employed to establish an early and precise diagnosis.

## Methods

### Clinical cohorts

We included PwPD from three distinct cohort studies into our analysis: (I) the Parkinson’s Progression Markers Initiative (PPMI, NCT04477785)^27^, which focuses on de-novo PwPD, (II) the French ICEBERG cohort study (NCT02305147) with early-stage PwPD, and (III) the Luxembourg Parkinson’s Study (LuxPARK, NCT05266872)^28^ comprising PwPD across all disease stages.

### PPMI

We analyzed 409 people with Parkinson’s Disease (PwPD) from the publicly available Parkinson’s Progression Markers Initiative (PPMI, NCT04477785) with clinical visits between 2011 and 2020. All PwPD had a clinical diagnosis of Parkinson’s Disease (PD) and a pathological dopamine transporter SPECT (DaTSCAN). Inclusion of PwPD in PPMI was restricted to untreated de-novo PwPD. In detail, inclusion was limited to PwPD with clinical diagnosis not more than two years before baseline visit, Hoehn & Yahr stage 0-2, no dopaminergic treatment at baseline visit, and PwPD with age > 30 years. Furthermore, we restricted our analysis to PwPD with at least one additional visit as we require longitudinal information. Informed consent to data collection and sharing was obtained from all PwPD by PPMI. Ethical guidelines on human data collection were adhered to. The PPMI project was approved by the Institutional Review Board or Independent Ethics Committee of all participating sites in Europe, including Attikon University Hospital (Greece), Hospital Clinic de Barcelona and Hospital Universitario Donostia (Spain), Innsbruck University (Austria), Paracelsus-Elena-Klinic Kassel/University of Marburg (Germany), Imperial College London (UK), Pitié-Salpêtrière Hospital (France), University of Salerno (Italy), and in the USA, including Emory University, Johns Hopkins University, University of Alabama at Birmingham, PD and Movement Disorders Center of Boca Raton, Boston University, Northwestern University, University of Cincinnati, Cleveland Clinic Foundation, Baylor College of Medicine, Institute for Neurodegenerative Disorders, Columbia University Medical Center, Beth Israel Medical Center, University of Pennsylvania, Oregon Health and Science University, University of Rochester, University of California at San Diego, and University of California, San Francisco.

### ICEBERG

We analyzed 154 PwPD from the ICEBERG cohort study (NCT02305147), an ongoing four-year observational study of PwPD with recent onset of PD conducted at the Paris Brain Institute (Institut du Cerveau-ICM, Pitié-Salpêtrière Hospital, Paris, France). Visits were performed between 2014 and 2022. PD was diagnosed according to UK Parkinson’s Disease Society Brain Bank criteria and PwPD with DaTSCANs showing no dopaminergic deficit were excluded. Inclusion was restricted to disease onset not more than three years before baseline visit. We further restricted our analysis to PwPD with at least two visits as we require longitudinal information. Informed consent was obtained and ethical guidelines were adhered to. ICEBERG received approval from the local ethical committee (IRBParis VI, RCB: 2014-A00725-42).

### LuxPARK

We analyzed 561 PwPD from the Luxembourg Parkinson’s Study (LuxPARK, NCT05266872), an ongoing observational study of all disease stages PwPD from Luxembourg and the Greater Region with up to four years follow up. Visits were performed between 2015 and 2022. PD was diagnosed according to UK Parkinson’s Disease Society Brain Bank criteria. We restricted our analysis to PwPD with at least two visits as we require longitudinal information. Informed consent was obtained and ethical guidelines were adhered to. LuxPARK was approved by the National Ethics Board in Luxembourg (CNER Ref: 201407/13).

### Estimating time shifts using a latent time joint mixed-effects model

To estimate the diagnostic delay of individual PwPD, we calculated how much each patient’s timescale is shifted from the average PD time course. Therefore, we modeled disease progression in each cohort as a linear process using a latent time joint mixed-effects model (LTJMM) as proposed from Li et al. ^16^.

In brief, LTJMM models linear progression of multiple clinical outcomes jointly over time, thereby estimating the clinical course of a mean PwPD and how individual PwPD are shifted from this average PD time course. Thereby, LTJMM includes random effects to accommodate for individual differences in progression speed and baseline values of clinical outcomes. These model-derived time shifts were used to align PwPD on a *common disease timescale* and reflect how early or late individual PwPD were diagnosed compared to an average PwPD.

The LTJMM is given by:

$${y}_{{ijk}}={x}_{i}{\beta }_{k}+{\gamma }_{k}\left({t}_{{ijk}}+{\delta }_{i}\right)+{\alpha }_{0{ik}}+{\alpha }_{1{ik}}{t}_{{ijk}}+{\epsilon }_{{ijk}}$$

Thereby, we denote y_ijk_ as outcome k observed at measurement j for an individual i. We account for age and sex differences by including age at diagnosis and sex as covariates x_i_ into the model with β_k_ as corresponding coefficient shared across all individuals. The coefficient ${\gamma }_{k}$ represents the mean slope of the cohort for each outcome k and is thereby shared across all individuals. We use the time since diagnosis as t_ijk_ and shift all measurements of an individual by a PwPD specific time shift δ_i_ shared across all outcomes. Additionally, we include random intercepts α_0ik_ and random slopes α_1ik_ for each individual and outcome. As usual, measurement errors ε_ijk_ and time shifts δ_i_ are both assumed to be drawn from normal distributions with a mean of zero. Random intercepts and slopes follow a multivariate normal distribution with mean of zero. Fitting was performed using a Markov chain Monte Carlo (MCMC) algorithm with 4 chains, 25000 iterations and 12500 warm up steps. Analyses were performed using the R packages ltjmm^29^ and rstan^30^.

Unified Parkinson’s Disease Rating Scale (UPDRS) I-IV, Postural Instability and Gait Dysfunction score (PIGD), Montreal Cognitive Assessment (MoCA) and Scales for Outcomes in Parkinson’s Disease-Autonomic Dysfunction (SCOPA) were used as outcomes and min-max-normalized on the theoretical range of the scores. MoCA scores were inverted to ensure positive slopes for all outcomes.

Convergence of MCMCs and normal distribution of parameter estimates were inspected manually. In addition, $\hat{R}$ statistics were calculated and ensured to be below 1.05.

To visualize and validate the effect of aligning PwPD on a *common disease timescale*, we inspected the distributions of Hoehn & Yahr (H&Y) stages which were not used for fitting the LTJMM model. Thereby, we observed a clearer separation of H&Y stages after applying LTJMM to the data and a stronger correlation between H&Y stages and the timescale (Fig. S8).

Further, we inspected the accuracy of our LTJMM approach in predicting outcomes at the next visit. Therefore, we re-trained LTJMM, but excluded the last measurement of all outcomes. Using this LTJMM model, we predicted these last measurements of all outcomes and calculated the coefficient of determination (R^2^) for these predictions. Thereby, we obtained reasonable R^2^ values: 55% (PPMI), 53% (ICEBERG), 61% (LuxPARK).

To analyze (I) the correlation of time shifts with age at diagnosis and (II) the time shift differences between female and male PwPD, we used a simplified LTJMM model without sex and age at diagnosis as covariates.

### Estimating clinical manifestation at the time when PD is diagnosed typically

Clinical symptoms are reported only at the time of study visits and are not available at the time of PD diagnosis. Therefore, we estimated the initial clinical manifestations at the time of PD diagnosis on the *common disease timescale*, i.e. at the time where ${t}_{i}+{\delta }_{i}=0$. This was achieved by applying the following steps to every outcome listed in Table S5: (I) aligning all measurements on the *common disease timescale*, (II) fitting linear, ordinal or binary mixed effect models on the measurement time series based on the scale of the outcome, (III) predicting the initial manifestation, i.e. the outcome at the time where ${t}_{i}+{\delta }_{i}=0$ on the *common disease timescale*. We restricted our analysis to outcomes with at least 2 measurements per PwPD as we require longitudinal data. Furthermore, we restricted our analysis to outcomes with data available for at least 30 PwPD.

To validate our model predictions, we compared the coefficient of determination with a null model for all three cohorts. More precisely, linear, ordinal and binary mixed effect models were fitted by leaving out the value of the first visit. R^2^ values of the first-visit predictions were calculated and compared with a null-model using the value of the second visit. For the null model, only visits being at least one year apart from the first visit were taken into account. A Wilcoxon signed rank-test was used to compare R^2^ values of the null model with the statistical models and indicated that the models outperformed the null model in all cohorts (*P* < 0.0001, Fig. S9).

The statistical models were fitted using the R packages lme4^31^ and ordinal^32^.

### Association of symptom domains with diagnostic delay

To assess the influence of clinical manifestations on the time of PD diagnosis, we analyzed the correlation of (I) patient-reported time to diagnosis with baseline clinical manifestations and (II) model-derived time shifts estimated by the LTJMM and initial clinical manifestations estimated by the statistical models.

Correlations between outcomes and the time variable (i.e., patient-reported time to diagnosis or model-derived time shifts) were calculated as Pearson, Kendall Tau-b and point-biserial correlation, depending on the scale of the outcome.

To allow a more comprehensive analysis and validation of the variety of outcomes captured across the three cohorts, we grouped 66 outcomes (including single questions, scores and sub-scores from questionnaires and clinical assessments) into 17 symptom domains (Table S5). The choice of the 17 symptom domains represents a trade-off between capturing most clinically relevant motor and non-motor symptoms and which outcomes had been assessed in the three cohorts.

To assess the correlation of clinical outcomes with the time variable in the defined symptom domains, we conducted a three-level meta-analysis with random effects for each symptom domain. Therefore, we first calculated an overall regression coefficient across all outcomes of a symptom domain per cohort. Subsequently, we calculated an overall regression coefficient estimate of the symptom domain across the three cohorts (see forest plots at end of the supplement). *P* values and 95% confidence intervals (CI) were corrected for multiple testing across the 17 symptom domains using the Benjamini-Hochberg procedure^33^. Meta analyses were performed using the R-package meta^34^.

### Motor phenotypes

PwPD were categorized as tremor dominant (TD) or postural instability and gait disturbance (PIGD) subtypes based on the ratio of the respective Unified Parkinson’s Disease Rating Scale (UPDRS) part III sub-scores^35^.

### Progression subtypes

We aimed to compare whether the rate of disease progression impacts how early or late PD is diagnosed by comparing two subtypes identified in a recent publication: a fast-progressing and a slow-progressing subtype^9^. In this recent analysis, we found that the fast-progressing subtype exhibited a more rapid progression of most symptoms, especially axial symptoms and non-motor symptoms. It was associated with worse response to dopaminergic treatment, higher levels of Alzheimer’s dementia pathology in cerebrospinal fluid, faster progression of neurodegeneration observed in DaTSCAN, and increased mortality.

Using the same approach as described in our previous publication, i.e. variational deep embedding with recurrence (VADER)^36^, we assigned all PwPD to one of these two subtypes.

### Statistical analyses

For comparison of cohort characteristics, the following tests were applied: Sex was compared using Fisher’s exact test, Hoehn & Yahr using the Kolmogorov-Smirnov test and all other characteristics were compared using the Mann-Whitney U test, thereby correcting for multiple testing using the Benjamini-Hochberg procedure^33^.

Correlations of patient-reported time since diagnosis and model-derived time shifts with age of diagnosis were calculated using Pearson correlation. Group differences regarding sex, family history, predominant side were carried out using t-test, Welch’s test or Mann-Whitney U test depending on the distribution of the data. Subgroup analyses for sex and age-specific subgroups were conducted for characteristics with significant associations in the overall analysis. The age-specific subgroups were defined by a median split on age of diagnosis covering PwPD from all three cohorts. All boxplots are displayed with a median line, box borders representing the interquartile range (IQR), whiskers extending to 1.5 times the IQR, and outliers depicted as diamonds beyond the whiskers.

All statistical tests were conducted as two-tailed tests with significance level 0.05 using the Python package SciPy^37^.

### Supplementary material

Details about datasets, additional figures and list of ICEBERG and NCER-PD members can be found in the supplement.

## Supplementary information

Supplemental Information

## Data Availability

As this study is a retrospective analysis, availability of the clinical data depends on the individual study groups (PPMI: www.ppmi-info.org, ICEBERG: marie.vidailhet@psl.aphp.fr, LuxPARK: rejko.krueger@uni.lu).

## Code availability

Relevant source code will be published at https://github.com/SCAI-BIO/PD-diagnostic-delay upon acceptance of the paper. Availability of the clinical data depends on the individual study groups.
