# Supplementary material for: Predictive modeling to uncover Parkinson’s disease characteristics that delay diagnosis
Source: NPJ Parkinsons Dis. 2025 Apr 2;11:64. doi: 10.1038/s41531-025-00923-2 (PMC11965517; doi:10.1038/s41531-025-00923-2)
Supplement: Supplementary file 1 — Supplemental Information [file 41531_2025_923_MOESM1_ESM.pdf]

Supplementary Data

Predictive Modeling to Uncover Parkinson's Disease Characteristics That Delay Diagnosis

Tom Hähnel[1,2], Tamara Raschka[1,3], Jochen Klucken[4,5,6], Enrico Glaab[4], Jean-Christophe Corvol[7], Björn H. Falkenburger*[2,8], Holger Fröhlich*[1,3]

1. Department of Bioinformatics, Fraunhofer Institute for Algorithms and Scientific Computing (SCAI), Sankt Augustin, Germany
2. Department of Neurology, Medical Faculty and University Hospital Carl Gustav Carus, TUD Dresden University of Technology, Dresden, Germany
3. Bonn-Aachen International Center for IT, University of Bonn, Bonn, Germany
4. Biomedical Data Science, Luxembourg Centre for Systems Biomedicine (LCSB), University of Luxembourg, Esch-sur-Alzette, Luxembourg
5. Luxembourg Institute of Health (LIH), Strassen, Luxembourg
6. Centre Hospitalier de Luxembourg (CHL), Luxembourg
7. Sorbonne Université, Paris Brain Institute – ICM, Inserm, CNRS, Assistance Publique Hôpitaux de Paris, Pitié-Salpêtrière Hospital, Department of Neurology, Paris, France
8. German Center for Neurodegenerative Diseases (DZNE), Dresden, Germany

* Both authors contributed equally

Corresponding author:
Dr. Tom Hähnel
Department of Bioinformatics
Fraunhofer Institute for Algorithms and Scientific Computing (SCAI)
Schloss Birlinghoven 1
53757 Sankt Augustin
Germany
Email: tom.haehnel@scai-extern.fraunhofer.de

Running title: Modeling Diagnostic Delay in Parkinson

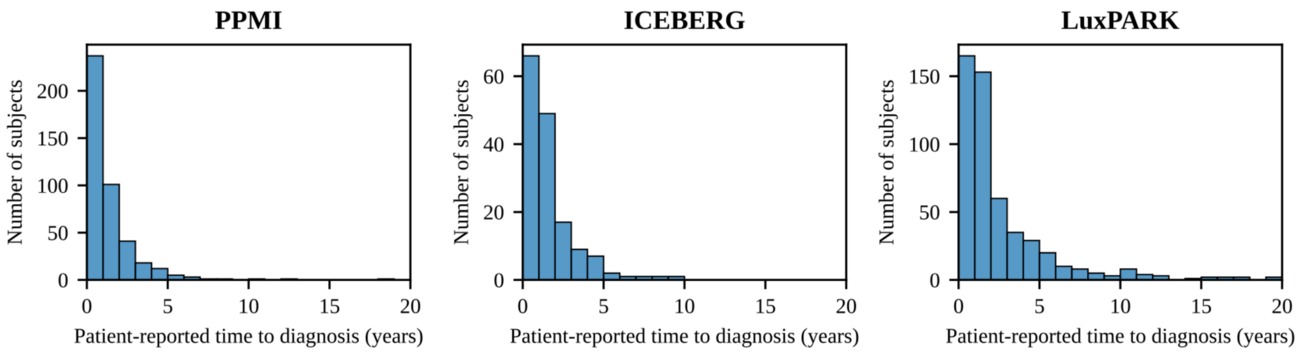

Figure S1: Distribution of patient-reported time to diagnosis
The histograms depict the distribution of patient-reported time to diagnosis for PPMI, ICEBERG and LuxPARK cohorts. Patient-reported time to diagnosis was defined as the time span between self-recognized occurrence of first motor symptom and PD diagnosis.

	**PPMI**	**ICEBERG**	**LuxPARK**
**Age at diagnosis**	ρ=-0.076<br>(P=0.12)	ρ=0.0017<br>(P=0.98)	ρ=0.035<br>(P=0.42)
**Sex**<br>(female/male)	d=0.0053<br>(P=0.37)	d=0.20<br>(P=0.19)	d=0.0081<br>(P=0.90)
**Family history of PD**<br>(no/yes)	d=-0.11<br>(P=0.31)	d=0.16<br>(P=0.93)	d=0.045<br>(P=0.87)
**Predominant side**<br>(left/right)	d=0.01<br>(P=0.46)	d=0.16<br>(P=0.13)	d=0.084<br>(P=0.91)

Table S1: Associations of demographic and clinical characteristics with patient-reported time to diagnosis
The relationships between age at PD diagnosis and patient-reported time to diagnosis were assessed using Pearson correlation with corresponding correlation coefficients and p-values being reported. Regarding sex, family history of PD, and predominant side, patient-reported times to diagnosis were compared between subgroups using Mann-Whitney U tests. Corresponding p-values and Cohen's D are reported. Positive Cohen's D values indicate a higher patient-reported time to diagnosis for female PwPD, negative family history, and PwPD with left predominant side.

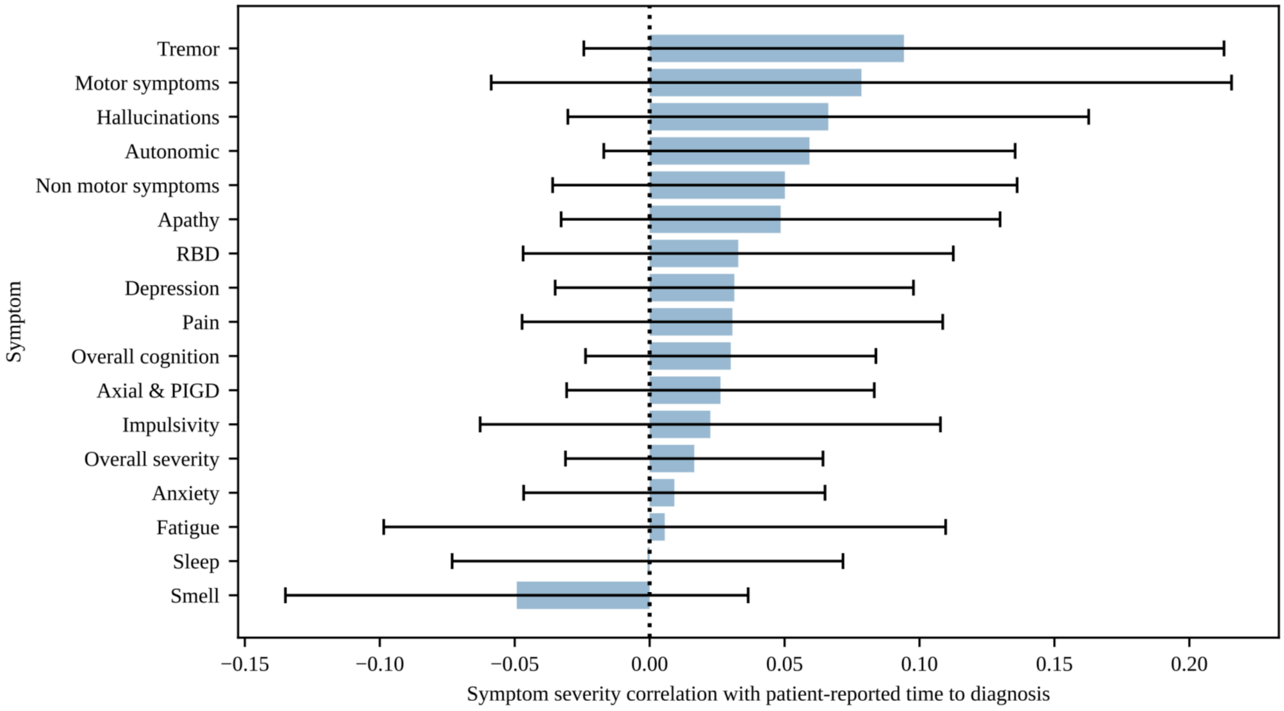

Figure S2 Correlations of baseline clinical characteristics with patient-reported time to diagnosis
The figure depicts the correlations between the severity of different symptom domains at baseline visit and the patient-reported time to diagnosis. Positive correlation coefficients mean that increased symptom severity is associated with a longer patient-reported time to diagnosis. The presented correlation coefficients are pooled estimates derived from several clinical scores from the PPMI and ICEBERG cohort (Table S5). The LuxPARK cohort was not included into this analysis as it included also advanced disease stage PwPD at baseline visit. Confidence intervals were corrected for multiple testing. P-values and correlation coefficients are also reported in Table S2.
Abbreviations: RBD: REM behavior sleep disorder, PIGD: postural instability and gait disturbance

Symptom	P value	Correlation coefficient
**Anxiety**	0.84	0.01
**Apathy**	0.44	0.05
**Autonomic**	0.27	0.06
**Axial & PIGD**	0.54	0.03
**Depression**	0.54	0.03
**Fatigue**	0.97	0.01
**Hallucinations**	0.41	0.07
**Impulsivity**	0.72	0.02
**Motor symptoms**	0.44	0.08
**Non motor symptoms**	0.44	0.05
**Overall cognition**	0.44	0.03
**Overall severity**	0.62	0.02
**Pain**	0.58	0.03
**RBD**	0.58	0.03
**Sleep**	0.98	0.00
**Smell**	0.44	-0.05
**Tremor**	0.27	0.09

Table S2: Correlations of baseline clinical characteristics with patient-reported time to diagnosis

The table reports the correlations of baseline clinical characteristics with patient-reported time to diagnosis. Positive correlation coefficients mean that increased symptom severity is associated with a longer patient-reported time to diagnosis. The presented correlation coefficients are pooled estimates derived from several clinical scores from the PPMI and ICEBERG cohort. The LuxPARK cohort was not included into this analysis as it included also advanced disease stage PwPD at baseline visit. Corresponding p-values were corrected for multiple testing.

Abbreviations: RBD: REM behavior sleep disorder, PIGD: postural instability and gait disturbance

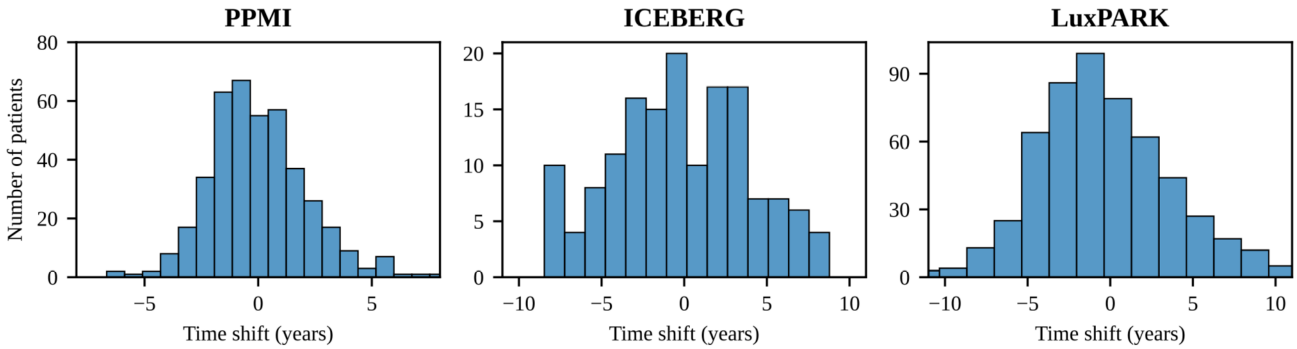

Figure S3 Distribution of model-derived time shifts across cohorts
The histograms depict the variation in model-derived time shifts for PPMI, ICEBERG and LuxPARK cohorts in relation to an average PwPD. Positive model-derived time shifts indicate that PD was diagnosed later than average.

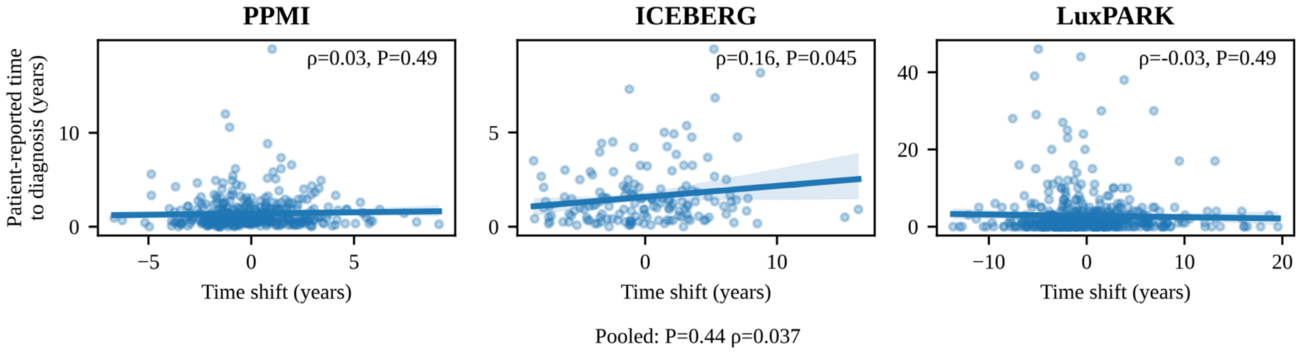

Figure S4: Correlation of patient-reported time to diagnosis with model-derived time shifts
Positive model-derived time shifts indicate that PD was diagnosed later than for an average PwPD in the corresponding cohort. Pearson correlation coefficients and corresponding p-values are shown for the correlations between patient-reported time to diagnosis and model-derived time shifts.

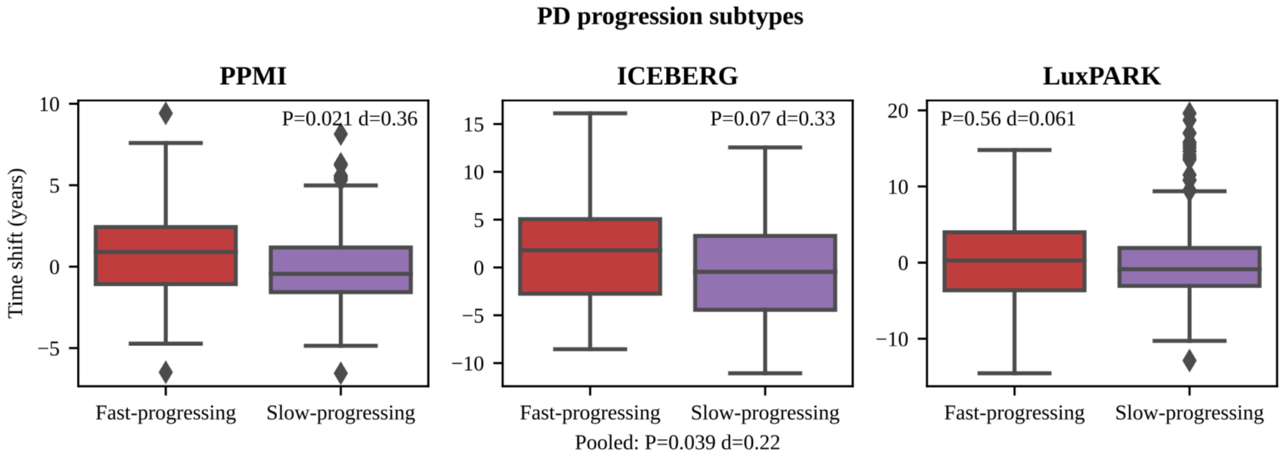

Figure S5: Distribution of model-derived time shifts in both PD progression subtypes

The figure depicts the differences in model-derived time shifts for the fast-progressing and slow-progressing PD subtypes identified in a previous publication. Positive model-derived time shifts indicate that PD was diagnosed later than the average patient in the corresponding cohort. Corresponding p-values of t-tests and Cohen's d are reported. Additionally, the pooled results from a meta-analysis across all three cohorts is shown below the plot.

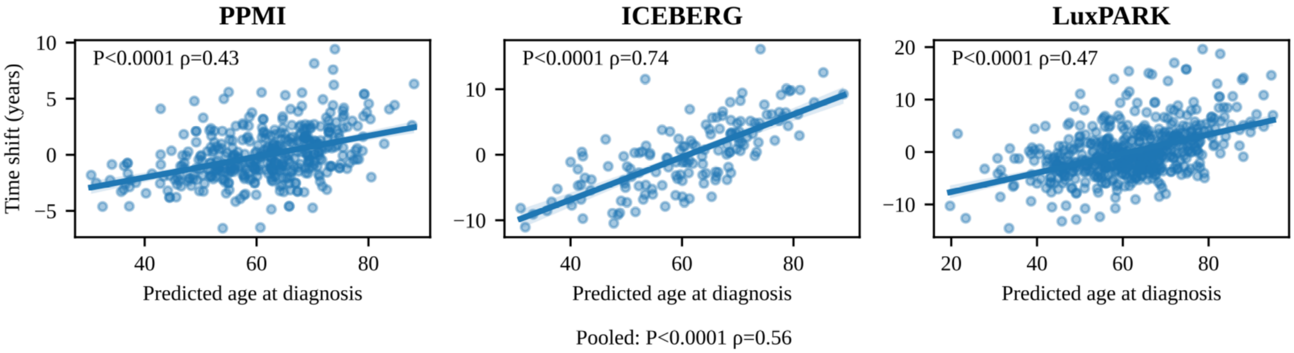

Figure S6: Correlation of estimated age at diagnosis with model-derived time shifts

The figure depicts the correlations between estimated age at the time of typical PD diagnosis (i.e. age at t=0 on the common disease timescale) and model-derived time shifts for PPMI, ICEBERG and LuxPARK. Positive model-derived time shifts indicate that PD was diagnosed later than average. Pearson correlation coefficients and corresponding p values are reported. The pooled result from a meta-analysis across all three cohorts was highly significant (P<0.0001, r=0.56).

		**PPMI**	**ICEBERG**	**LuxPARK**	**Pooled estimate**
**Predicted age at diagnosis**		**ρ=0.43<br>(P<0.0001)**	**ρ=0.74<br>(P<0.0001)**	**ρ=0.47<br>(P<0.0001)**	**ρ=0.56<br>(P<0.0001)**
	**female**	**ρ=0.25<br>(P=0.0037)**	**ρ=0.68<br>(P<0.0001)**	**ρ=0.46<br>(P<0.0001)**	**ρ=0.48<br>(P=0.0015)**
	**male**	**ρ=0.51<br>(P<0.0001)**	**ρ=0.77<br>(P<0.0001)**	**ρ=0.48<br>(P<0.0001)**	**ρ=0.60<br>(P<0.0001)**
**Sex**<br>(female/male)		d=0.099<br>(P=0.34)	d=0.13<br>(P=0.45)	d=-0.079<br>(P=0.38)	d=0.022<br>(P=0.76)
**Family history of PD**<br>(no/yes)		d=-0.079<br>(P=0.49)	d=0.12<br>(P=0.52)	d=0.041<br>(P=0.67)	d=-0.0084<br>(P=0.90)
**Predominant side**<br>(left/right)		d=-0.099<br>(P=0.32)	d=-0.015<br>(P=0.93)	d=0.18<br>(P=0.19)	d=0.0069<br>(P=0.94)
**Motor phenotype**<br>(TD/PIGD)		d=0.24<br>(P=0.20)	d=0.15<br>(P=0.83)	d=0.21<br>(P=0.20)	**d=0.21<br>(P=0.0047)**
	**young (<62.2 years)**	d=0.14<br>(P=0.99)	d=0.24<br>(P=0.92)	d=0.08<br>(P=0.69)	d=0.12<br>(P=0.25)
	**old (>= 62.2 years)**	d=0.36<br>(P=0.088)	d=0.01<br>(P=0.75)	d=0.31<br>(P=0.15)	**d=0.30<br>(P=0.0049)**
	**female**	d=0.05<br>(P=0.37)	d=-0.35<br>(P=0.07)	d=0.16<br>(P=0.59)	d=0.05<br>(P=0.69)
	**male**	**d=0.33<br>(P=0.018)**	d=0.49<br>(P=0.22)	d=0.24<br>(P=0.057)	**d=0.29<br>(P=0.002)**
**Progression subtype**<br>(fast-progressing/slow-progressing)		**d=0.36<br>(P=0.021)**	d=0.33<br>(P=0.07)	d=0.061<br>(P=0.56)	**d=0.22<br>(P=0.039)**
	**young (<62.2 years)**	d=0.23<br>(P=0.32)	**d=0.86<br>(P=0.0015)**	d=0.052<br>(P=0.78)	d=0.34<br>(P=0.14)
	**old (>= 62.2 years)**	d=0.3<br>(P=0.12)	d=-0.26<br>(P=0.33)	d=0.019<br>(P=0.89)	d=0.066<br>(P=0.62)
	**female**	d=-0.061<br>(P=0.85)	d=0.20<br>(P=0.54)	d=0.28<br>(P=0.11)	d=0.17<br>(P=0.20)
	**male**	**d=0.54<br>(P=0.00043)**	d=0.37<br>(P=0.096)	d=-0.029<br>(P=0.82)	d=0.28<br>(P=0.14)

Table S3: Associations of demographic and clinical characteristics with model-derived time shifts
The relationships between predicted age at PD diagnosis and model-derived time shifts were assessed using Pearson correlation with corresponding p-values being reported. Regarding sex, family history of PD, predominant side, progression subtype, and motor phenotype, model-derived time shifts were compared between subgroups using t-tests or Welch's test depending on the distribution of the data. Corresponding p-values and Cohen's D are shown. Thereby, positive Cohen's D values indicate a higher model-derived time shift for female PwPD, negative family history, PwPD with left predominant side, TD phenotype, and the fast-progressing PD subtype. Pooled estimates were calculated from meta-analyses across the three cohorts. Analyses of sex and predicted age at diagnosis were carried out using the LTJMM model without age at diagnosis and sex as covariates. Analyses of family history, motor phenotype, progression subtype and predominant side associations were carried out using the LTJMM model including age at diagnosis and sex as covariates. Significant results are indicated in bold. Subgroup analyses were carried out for characteristics with a significant pooled effect.
Abbreviations: TD: tremor-dominant, PIGD: postural instability and gait disturbance.

Symptom domain	All PwPD	Male PwPD	Female PwPD	Young PwPD (Age at diagnosis <= 62.2 years, median split)	Old PwPD (Age at diagnosis > 62.2 years, median split)	Early onset PD (Age at diagnosis < 50 years)	Late onset PD (Age at diagnosis >= 50 years)
**Anxiety**	**0.13 (P=0.0043)**	**0.12 (P=0.005)**	**0.14 (P=0.019)**	**0.17 (P=0.00036)**	**0.11 (P=0.025)**	0.14 (P=0.055)	0.1 (P=0.066)
**Apathy**	0.02 (P=0.32)	0.03 (P=0.41)	0.0 (P=0.99)	0.05 (P=0.23)	-0.04 (P=0.36)	0.04 (P=0.79)	0.0 (P=0.88)
**Autonomic**	**0.1 (P=0.0019)**	**0.1 (P=0.014)**	**0.11 (P=0.0051)**	**0.14 (P=0.015)**	0.05 (P=0.098)	0.11 (P=0.29)	**0.09 (P=0.0081)**
**Overall cognition**	0.0 (P=0.88)	0.0 (P=0.95)	0.01 (P=0.92)	0.03 (P=0.42)	**-0.07 (P=0.022)**	0.01 (P=0.99)	-0.01 (P=0.67)
**Depression**	**0.1 (P=0.0004)**	**0.11 (P=0.0005)**	**0.09 (P=0.011)**	**0.1 (P=0.025)**	0.05 (P=0.23)	**0.12 (P=0.033)**	**0.09 (P=0.0018)**
**Fatigue**	**0.13 (P=0.012)**	0.11 (P=0.084)	0.21 (P=0.13)	0.2 (P=0.088)	0.08 (P=0.36)	0.15 P=0.17)	0.12 (P=0.066)
**Hallucinations**	-0.05 (P=0.092)	-0.02 (P=0.7)	-0.07 (P=0.26)	-0.06 (P=0.088)	-0.07 (P=0.19)	-0.08 (P=0.38)	-0.04 (P=0.14)
**Impulsivity**	0.07 (P=0.12)	0.02 (P=0.78)	**0.19 (P=0.019)**	0.11 (P=0.097)	0.07 (P=0.36)	0.11 (P=0.5)	0.07 (P=0.17)
**Motor symptoms**	-0.05 (P=0.23)	-0.05 (P=0.41)	-0.04 (P=0.3)	0.0 (P=0.97)	**-0.13 (P=0.00014)**	-0.0 (P=0.99)	-0.07 (P=0.11)
**Non motor symptoms**	**0.15 (P=0.0006)**	**0.14 (P=0.0082)**	**0.14 (P=0.0077)**	**0.23 (P=0.0007)**	0.04 (P=0.36)	0.18 (P=0.11)	**0.13 (P=0.0046)**
**Overall severity**	-0.04 (P=0.28)	-0.055 (P=0.3)	-0.07 (P=0.14)	0.01 (P=0.94)	**-0.14 (P<0.0001)**	0.02 (P=0.91)	-0.05 (P=0.14)
**Pain**	**0.08 (P=0.0085)**	0.07 (P=0.21)	**0.11 (P=0.019)**	**0.12 (P=0.007)**	0.08 (P=0.12)	**0.15 (P=0.033)**	0.06 (P=0.11)
**Axial & PIGD**	**-0.12 (P=0.0004)**	**-0.14 (P<0.0001)**	**-0.13 (P=0.011)**	-0.06 (P=0.069)	**-0.19 (P<0.0001)**	-0.09 (P=0.38)	**-0.14 (P=0.00018)**
**Sleep**	**0.06 (P=0.0043)**	0.04 (P=0.21)	**0.12 (P=0.0029)**	**0.09 (P=0.0017)**	0.01 (P=0.77)	**0.14 (P=0.033)**	0.04 (P=0.11)
**RBD**	0.06 (P=0.28)	0.07 (P=0.27)	0.05 (P=0.42)	0.03 (P=0.82)	0.04 (P=0.47)	0.1 (P=0.5)	0.06 (P=0.17)
**Smell**	-0.03 (P=0.79)	-0.05 (P=0.68)	-0.06 (P=0.57)	-0.08 (P=0.42)	-0.04 (P=0.74)	-0.17 (P=0.28)	-0.02 (P=0.78)
**Tremor**	-0.03 (P=0.75)	0.03 (P=0.78)	-0.12 (P=0.13)	-0.05 (P=0.48)	-0.04 (P=0.53)	-0.01 (P=0.99)	-0.04 (P=0.46)

Table S4: Correlations between initial clinical characteristics and model-derived time shifts for PD subgroups

The table reports the correlations of estimated initial clinical characteristics at the point of a typical PD diagnosis with model-derived time shifts. Positive correlation coefficients mean that increased symptom severity is associated with a PD diagnosis later than average. The presented correlation coefficients are pooled estimates derived from the three cohorts and several clinical scores. Corresponding p-values were corrected for multiple testing. Significant correlations are indicated bold. Results are shown for the overall PD cohorts, a sex-specific subgroup analysis, a subgroup analysis based on a median split of age at diagnosis, and a subgroup analysis based on the official MDS definition of early onset PD and late onset PD.

Abbreviations: RBD: REM behavior sleep disorder, PIGD: postural instability and gait disturbance

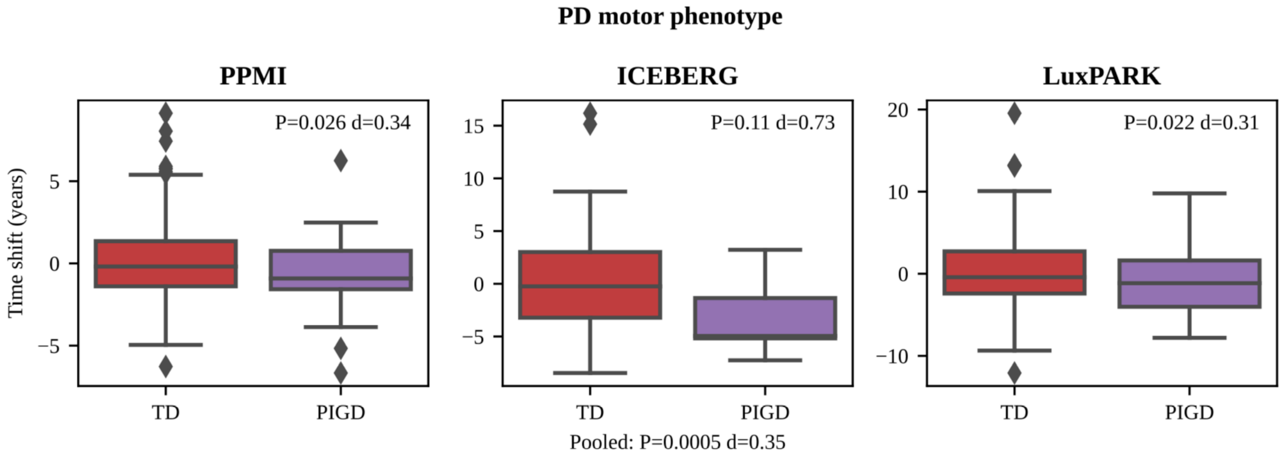

Figure S7: Distribution of model-derived time shifts for PD motor phenotypes

The figure depicts the differences in model-derived time shifts for the TD and PIGD motor phenotype. Positive model-derived time shifts indicate that PD was diagnosed later than the average patient in the corresponding cohort. Corresponding p-values of t-tests and Cohen's d are reported. Additionally, the pooled results from a meta-analysis across all three cohorts is shown below the plot.

Abbreviations: TD: tremor-dominant, PIGD: postural instability and gait disturbance

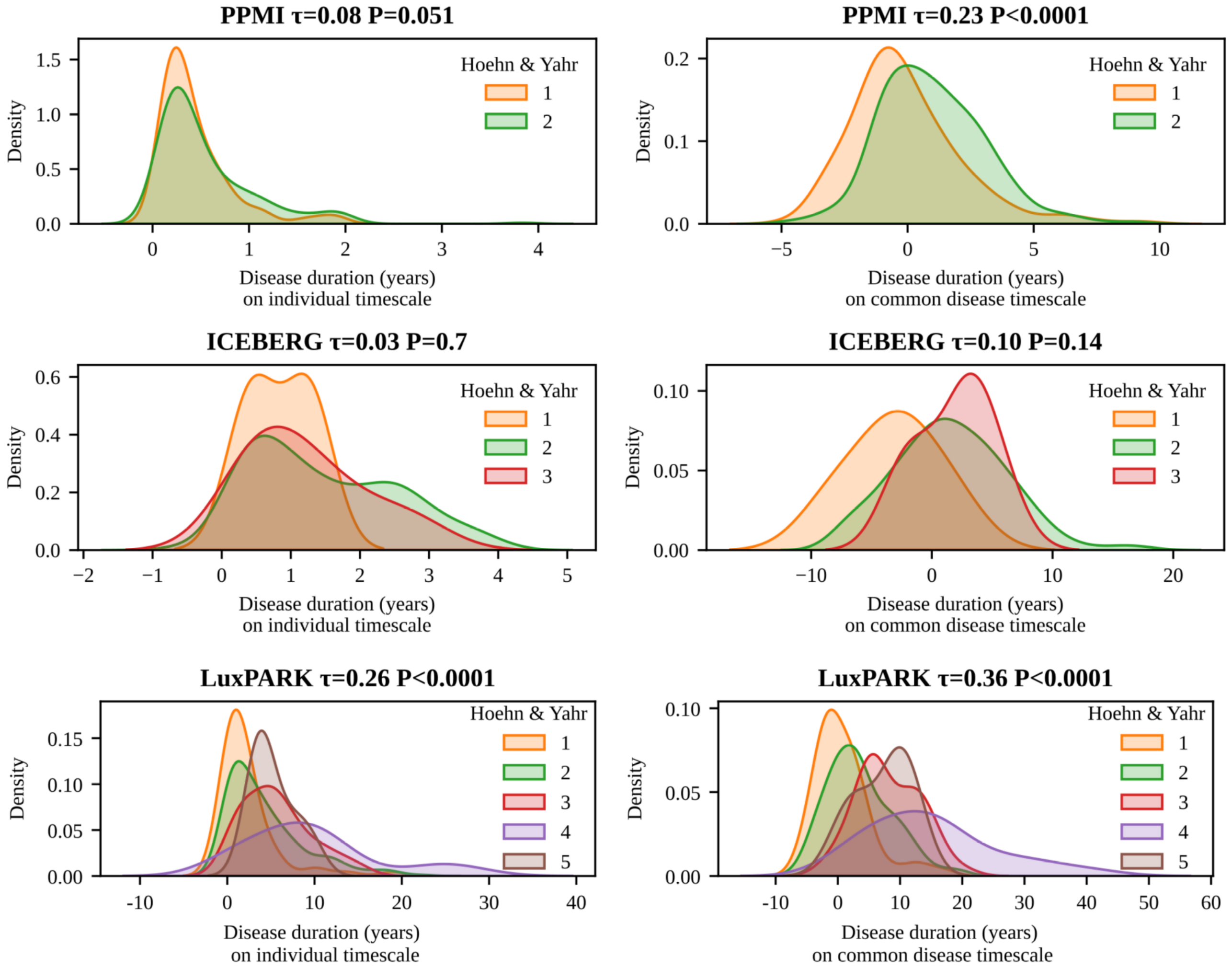

Figure S8: Effect of time-aligning PwPD on distributions of H&Y stages
H&Y baseline distributions from PPMI, ICEBERG and LuxPARK are depicted as kernel density estimation plots. On the left side, H&Y stages are plotted against the original timescale. On the right side, H&Y stages are plotted against the common disease timescale calculated from the LTJMM. Correlation of H&Y stages with the timescale are reported by Kendall tau-b correlation coefficients and corresponding p-values. Thereby, stronger correlations are observed after aligning PwPD on the common disease timescale.
Abbreviations: H&Y: Hoehn&Yahr, LTJMM: latent time joint mixed-effects model

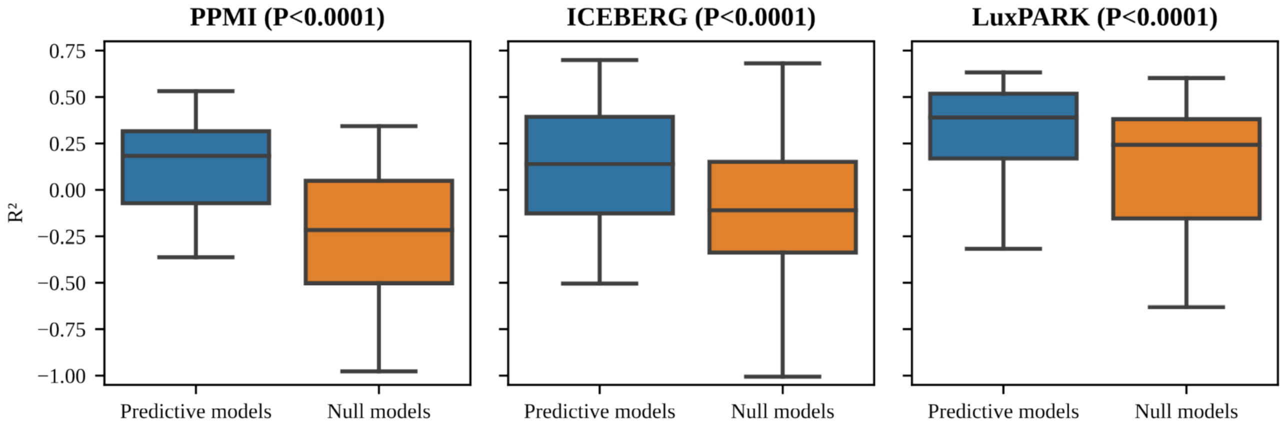

Figure S9: Predictive performance of statistical models to predict initial symptoms

Predictive performance of statistical models compared to a null model for the three cohorts. Linear, ordinal and binary mixed effect models were fitted to the longitudinal data of the outcomes listed in Table S5, thereby leaving out the value of the first visit. R^2 *values of the first-visit predictions were calculated and compared with a null-model using the value of the second visit. For the null model, only visits being at least one year apart from the first visit were taken into account. Corresponding p-values were calculated using a Wilcoxon signed-rank test.*

Symptom domain	Outcome	Definition/calculation of the outcome
Anxiety	NMSQ Anxiety	NMSQ item 17
	HADS anxiety	HADS anxiety sub-score
	STA	STA sum score
	PDQ39 Anxiety	PDQ39 item 21
	UPDRS I Anxiety	UPDRS I item 4
Apathy	DAS	DAS sum score
	SAS	SAS sum score
	UPDRS I Apathy	UPDRS I item 5
Autonomic symptoms	NMSQ Autonomic	NMSQ sum of items 4, 5, 6, 7, 8, 9, 19, 20, 28
	SCOPA-AUT	SCOPA sum score
	UPDRS I Autonomic	UPDRS I sum of items 10, 11, 12
Overall Cognition	MATTIS	MATTIS sum score
	MMSE	MMSE sum score
	MoCA	MoCA sum score
	SIQCDE	Short IQCODE score sum score
	FAB	FAB sum score
	PDQ39 Cognition	PDQ39 sum of items 31, 32
	UPDRS I Cognition	UPDRS I item 1
	NMSQ Cognition	NMSQ sum of items 12, 15
Depression	BDI	BDI sum score
	GDS	GDS sum score
	HADS depression	HADS depression sub-score
	PDQ39 Depression	PDQ39 sum of items 17, 18, 19, 20, 22
	NMSQ Depression	NMSQ sum of items 13, 16
	UPDRS I Depression	UPDRS 1 item 3
Fatigue	UPDRS I Fatigue	UPDRS 1 item 13
Hallucinations	NMSQ Hallucination	NMSQ sum of items 14, 30
	UPDRS I Hallucinations	UPDRS 1 item 2
Impulsivity	QUIP	QUIP sum score
	QUIP-RS	QUIPRS sum score
Motor symptoms (overall)	PDQ39 ADL	PDQ39 ADL sub-score
	Pegboard	PEGBoard sum of: average of left hand, right hand and both hands
	UPDRS II	UPDRS II sum score
	UPDRS III off	UPDRS III sum score (OFF only)
	UPDRS IV	UPDRS IV sum score
Non motor symptoms (overall)	NMSQ	NMSQ sum score
	UPDRS I	UPDRS I sum score
Overall disease severity	UPDRS I-III off	UPDRS I, II, III sum (OFF only)
	FAQ	FAQ sum score
	PDQ39	PDQ39 sum score
	SEADL	SEADL score
	H&Y	Hoehn & Yahr

**Symptom domain**	**Outcome**	**Definition/calculation of the outcome**
	CGIS	CGI-S score
Pain	NMSQ Pain	NMSQ item 10
	PDQ39 Pain	PDQ39 sum of items 37, 38
	UPDRS I Pain	UPDRS 1 item 9
Axial & PIGD symptoms	UPDRS III axial off	UPDRS III axial score (OFF only)
	FOGAC	FOGAC sum score
	FOGQ	FOGQ sum score
	GABS Examination	GABS sum of items 8-24
	GABS Questionnaire	GABS sum of items 1-7
	NFOGQ	NFOGQ sum score
	PDQ39 Mobility	PDQ39 mobility sub-score
	PIGD off	PIGD score (OFF only)
	TUG	Timed Up and Go time
Sleep (general)	ESS	ESS sum score
	PDSS	PDSS sum score
	UPDRS I Sleep	UPDRS I sum of items 7, 8
	NMSQ Sleep	NMSQ sub of items 22, 23
RBD Sleep	RBD-HK	RBD-HK sum score
	RBD-SQ	RBD-SQ sum score
	NMSQ RBD	NMSQ sum of items 24, 25
Smell	NMSQ Smell	NMSQ item 2
	Sniffin Test	Sniffin Test score
	UPSIT	UPSIT sum score
Tremor	TD off	TD score (OFF only)

Table S5: Construction of symptom domains

Abbreviations: BDI: Beck Depression Inventory, CGIS: Clinical Global Impression-Severity, DAS: Dimensional Apathy Scale, ESS: Epworth Sleepiness Scale, FAB: Frontal Assessment Battery, FAQ: Functional Activities Questionnaire, FOGAC: Freezing of Gait AC, FOGQ: Freezing of Gait Questionnaire, GABS: Clinical Gait and Balance Scale, GDS: Geriatric Depression Scale, H&Y: Hoehn & Yahr scale, HADS: Hospital Anxiety and Depression Scale, MATTIS: Mattis Dementia Rating Scale, MMSE: Mini Mental Status Examination, MOCA: Montreal Cognitive Assessment, NFOGQ: New Freezing of Gait Questionnaire, NMSQ: Non-Motor Symptoms Questionnaire, PDQ39: Parkinson's Disease Questionnaire-39, PDSS: Parkinson's Disease Sleep Scale, PIGD: Postural Instability and Gait Disorder score, QUIP: Questionnaire for Impulsive-Compulsive Disorders, QUIP-RS: QUIP-Rating Scale, RBD-HK: REM Sleep Behavior Disorder Questionnaire-Hong Kong, RBD-SQ: REM Sleep Behavior Disorder Screening Questionnaire, SAS: Starkstein Apathy Scale, SCOPA-AUT: Scales for Outcomes in Parkinson's Disease-Autonomic Dysfunction, SEADL: Schwab and England Activities of Daily Living Scale, SIQCDE: Short Informant Questionnaire on Cognitive Decline in the Elderly, STA: State-Trait Anxiety Inventory, TD: Tremor Dominance Score, TUG: Timed Up and Go, UPDRS: MDS-Unified Parkinson's Disease Rating Scale, UPSIT: University of Pennsylvania Smell Identification Test

Forest plots for correlation of baseline symptom domains with patient-reported time to diagnosis

Forest plot for domain Axial & PIGD

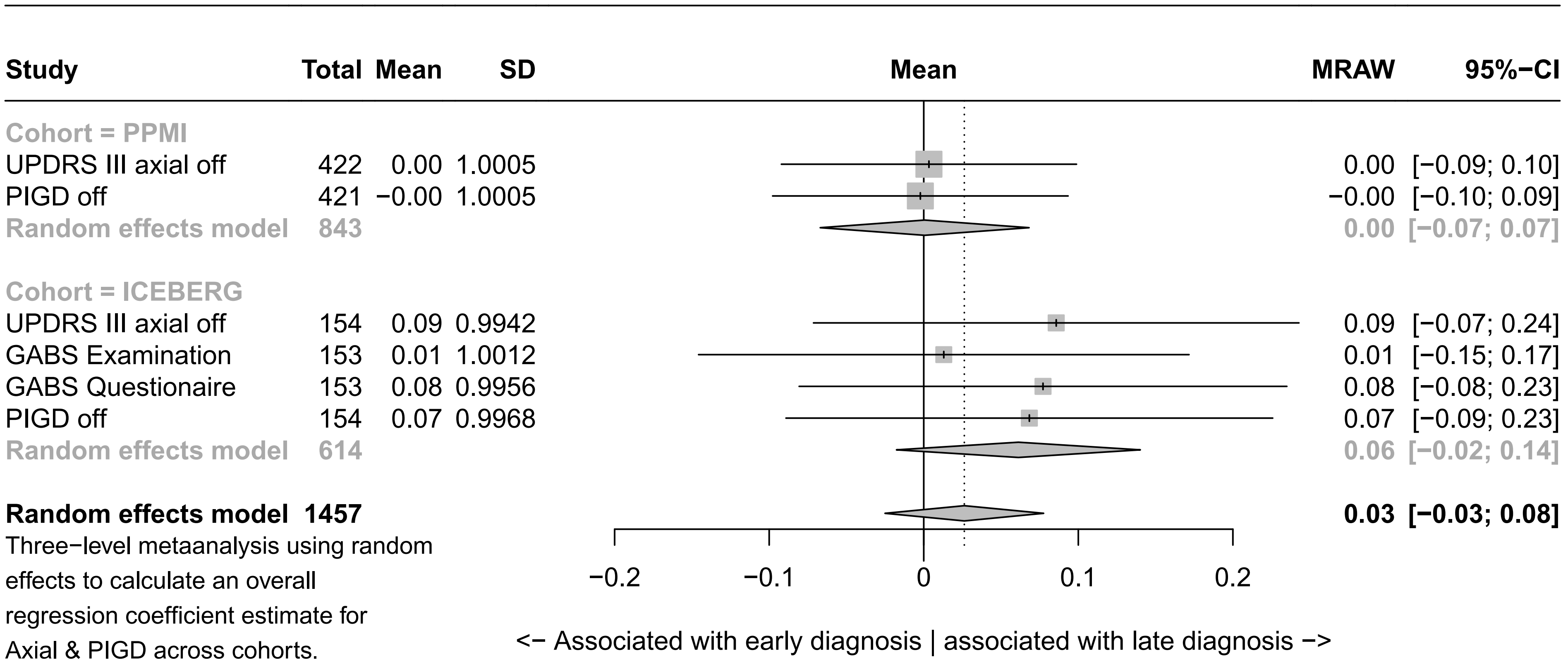

Three−level metaanalysis using random effects to calculate an overall regression coefficient estimate for Axial & PIGD across cohorts. The dashed line indicates the overall mean estimate. The solid line indicates no effect.

Forest plot for domain Overall severity

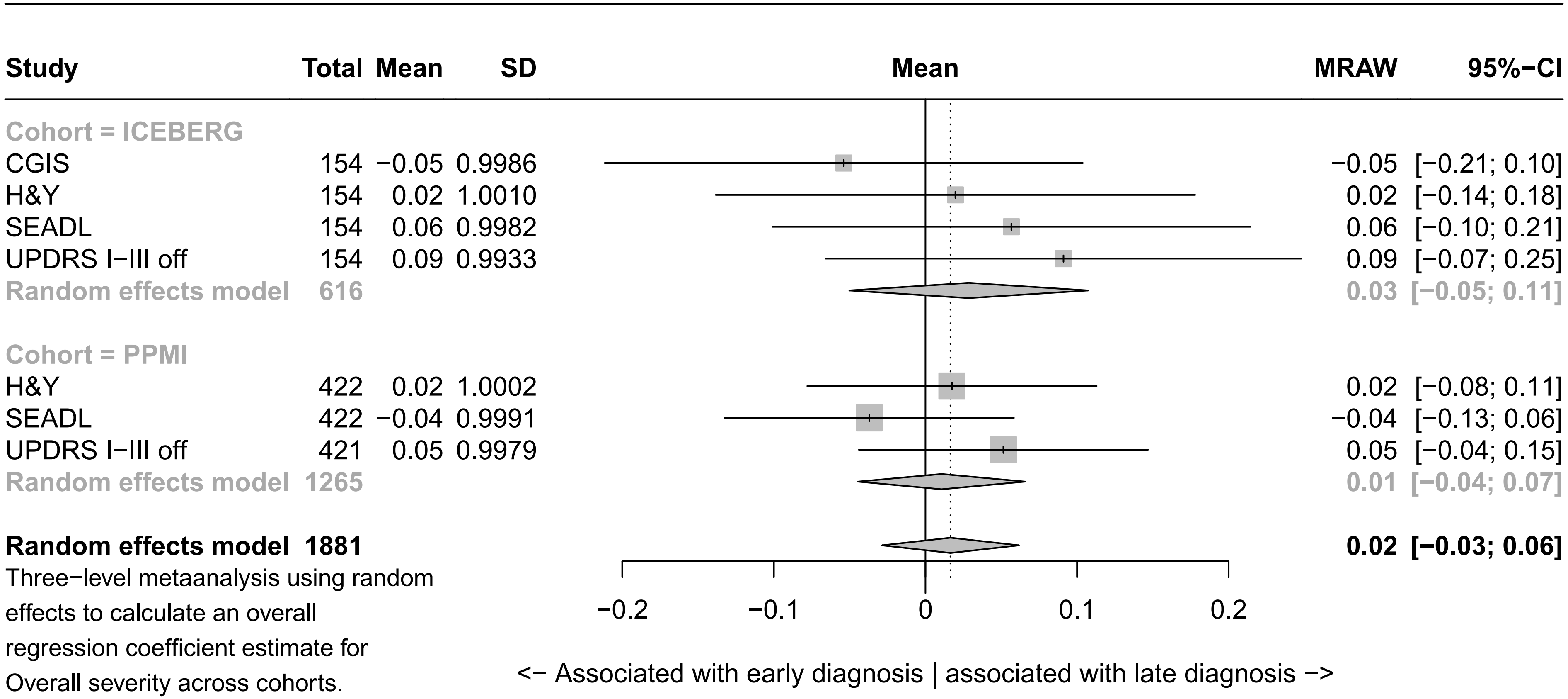

Three−level metaanalysis using random effects to calculate an overall regression coefficient estimate for Overall severity across cohorts. The dashed line indicates the overall mean estimate. The solid line indicates no effect.

Forest plot for domain Apathy

Study	Total	Mean	SD	MRAW	95%−CI
**Cohort = ICEBERG**					
DAS	108	0.18	0.9700	0.18	[−0.00; 0.36]
SAS	154	0.02	1.0011	0.02	[−0.14; 0.17]
UPDRS I Apathy	154	0.06	0.9976	0.06	[−0.10; 0.22]
**Random effects model**	**416**			**0.08**	**[−0.02; 0.17]**
**Cohort = PPMI**					
UPDRS I Apathy	422	0.02	1.0001	0.02	[−0.08; 0.11]
**Random effects model**	**838**			**0.05**	**[−0.02; 0.12]**

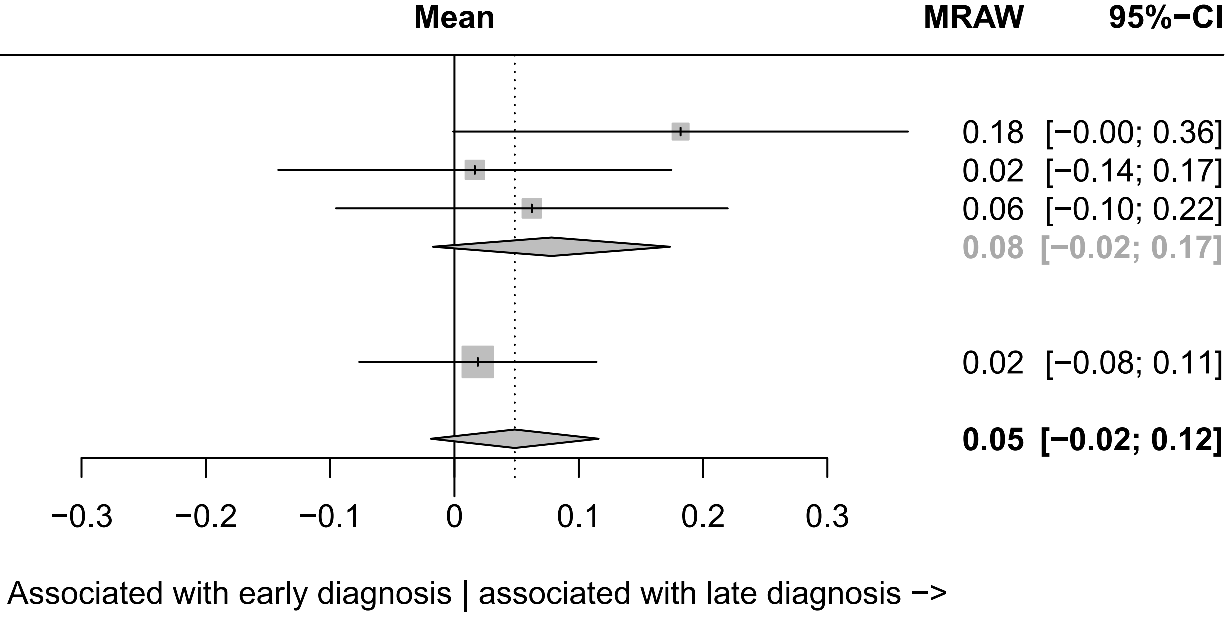

Three−level metaanalysis using random effects to calculate an overall regression coefficient estimate for Apathy across cohorts.
The dashed line indicates the overall mean estimate. The solid line indicates no effect.

Forest plot for domain Sleep

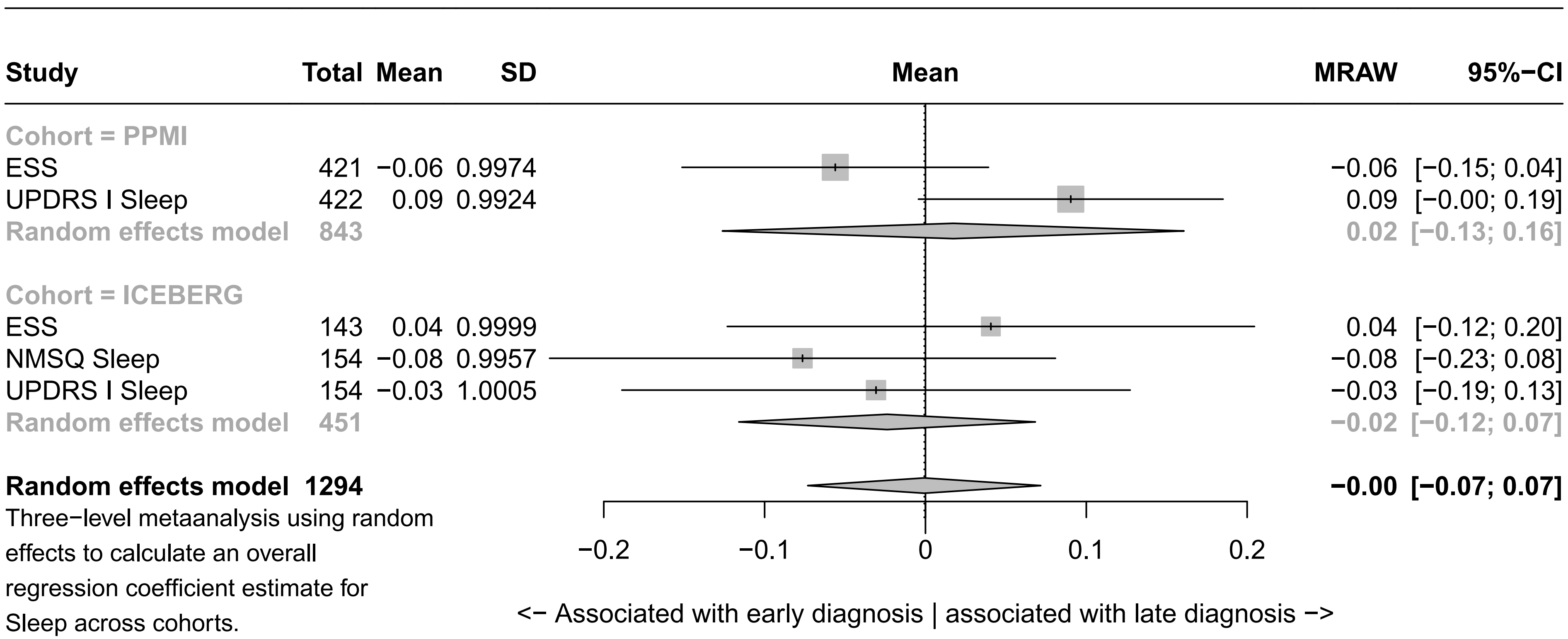

Three−level metaanalysis using random effects to calculate an overall regression coefficient estimate for Sleep across cohorts.
The dashed line indicates the overall mean estimate. The solid line indicates no effect.

Forest plot for domain Overall cognition

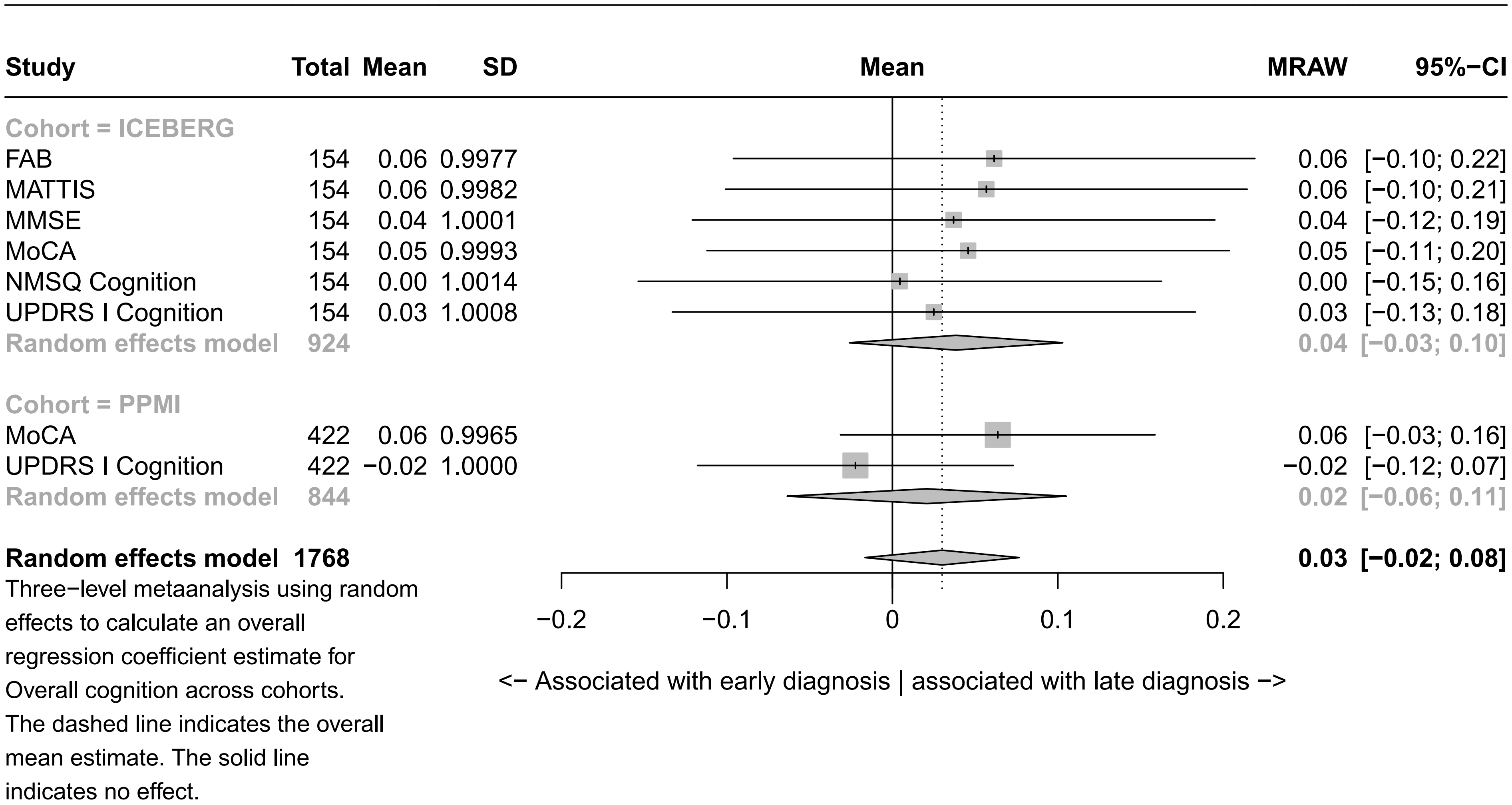

Forest plot for domain Depression

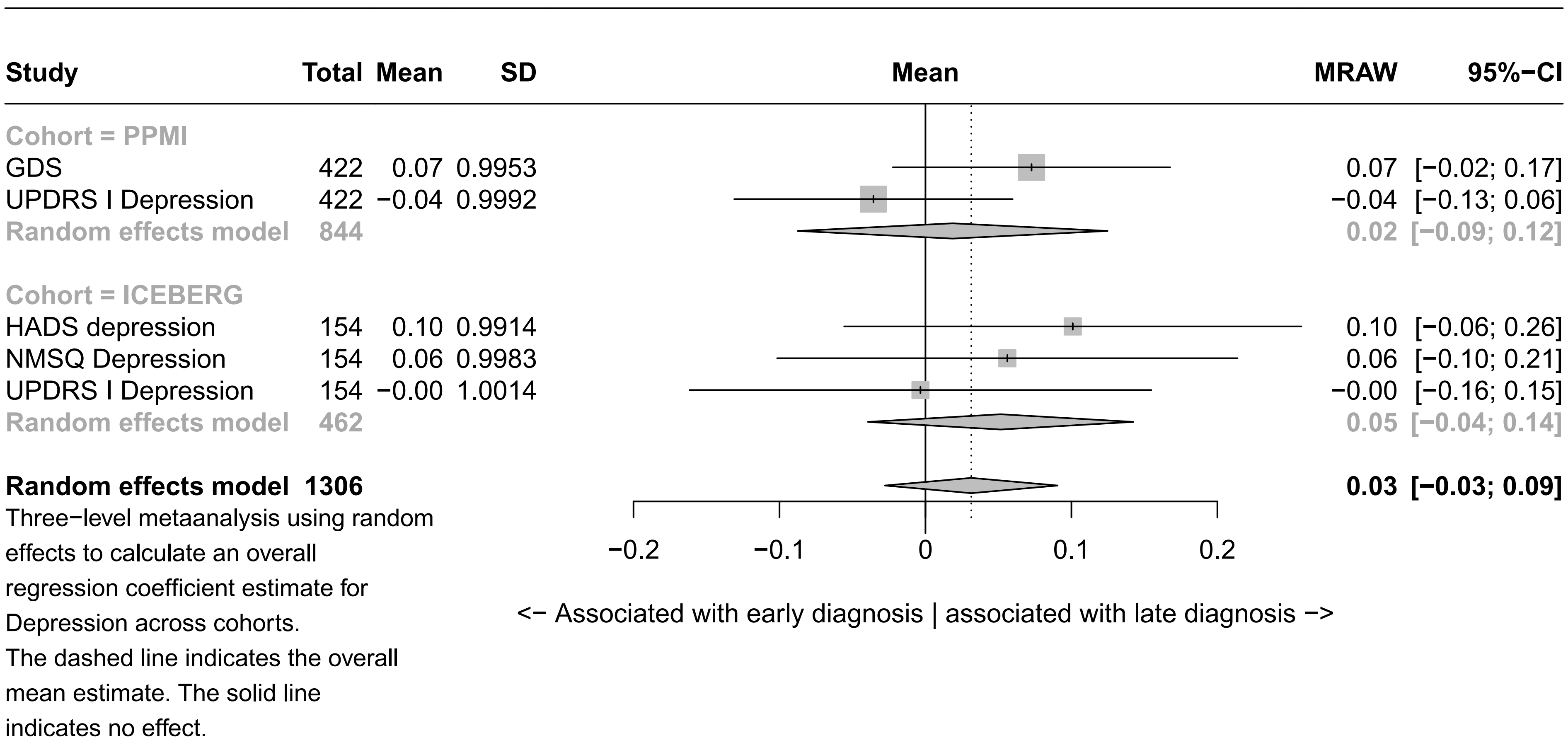

Forest plot for domain Anxiety

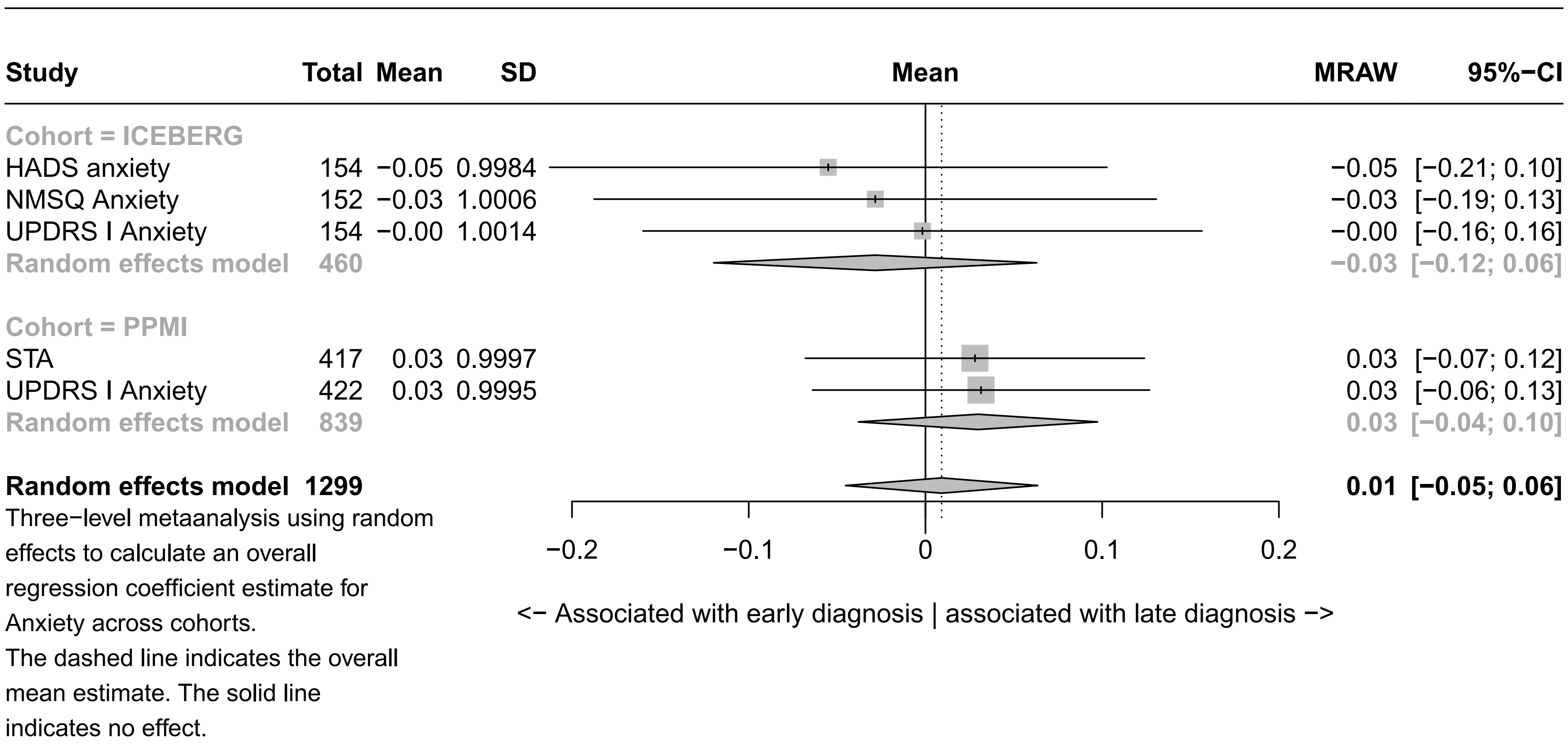

Forest plot for domain Non motor symptoms

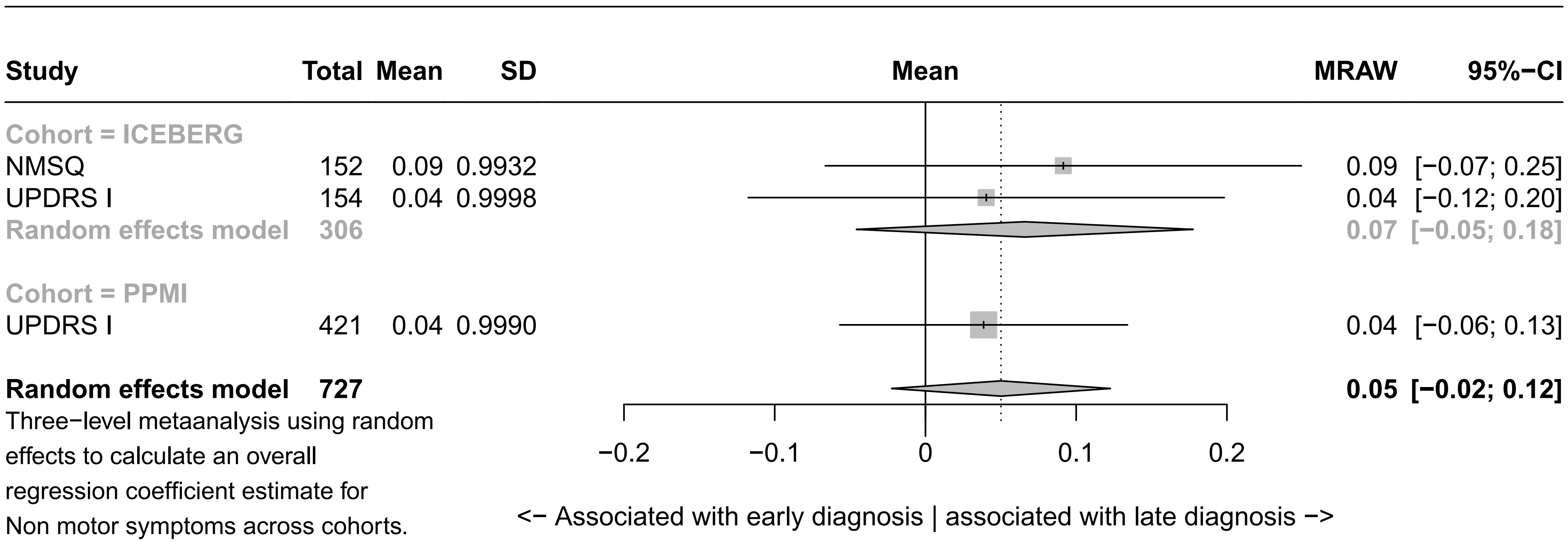

Three−level metaanalysis using random effects to calculate an overall regression coefficient estimate for Non motor symptoms across cohorts. The dashed line indicates the overall mean estimate. The solid line indicates no effect.

Forest plot for domain Autonomic

Study	Total	Mean	SD	MRAW	95%−CI
Cohort = ICEBERG					
NMSQ Autonomic	154	0.14	0.9821	0.14	[−0.01; 0.30]
SCOPA−AUT	153	0.08	0.9958	0.08	[−0.08; 0.23]
UPDRS I Autonomic	154	0.11	0.9894	0.11	[−0.05; 0.27]
Random effects model	461			0.11	[0.02; 0.20]
Cohort = PPMI					
SCOPA−AUT	411	0.01	1.0005	0.01	[−0.09; 0.10]
UPDRS I Autonomic	422	0.05	0.9975	0.05	[−0.04; 0.15]
Random effects model	833			0.03	[−0.04; 0.10]
**Random effects model**	**1294**			**0.06**	**[0.00; 0.11]**

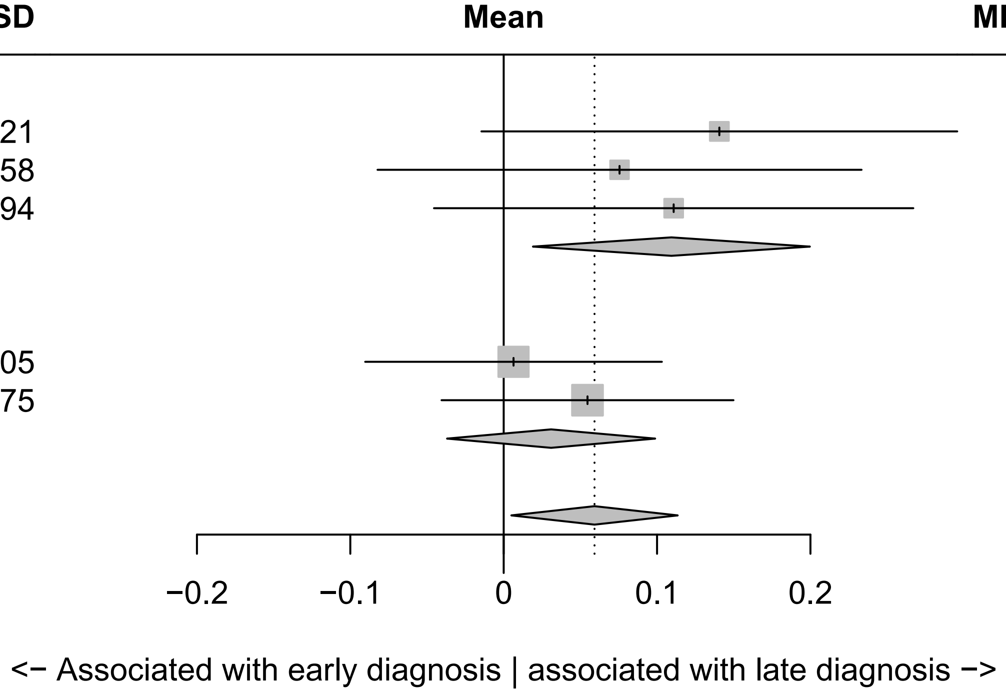

Three−level metaanalysis using random effects to calculate an overall regression coefficient estimate for Autonomic across cohorts.
The dashed line indicates the overall mean estimate. The solid line indicates no effect.

Forest plot for domain Hallucinations

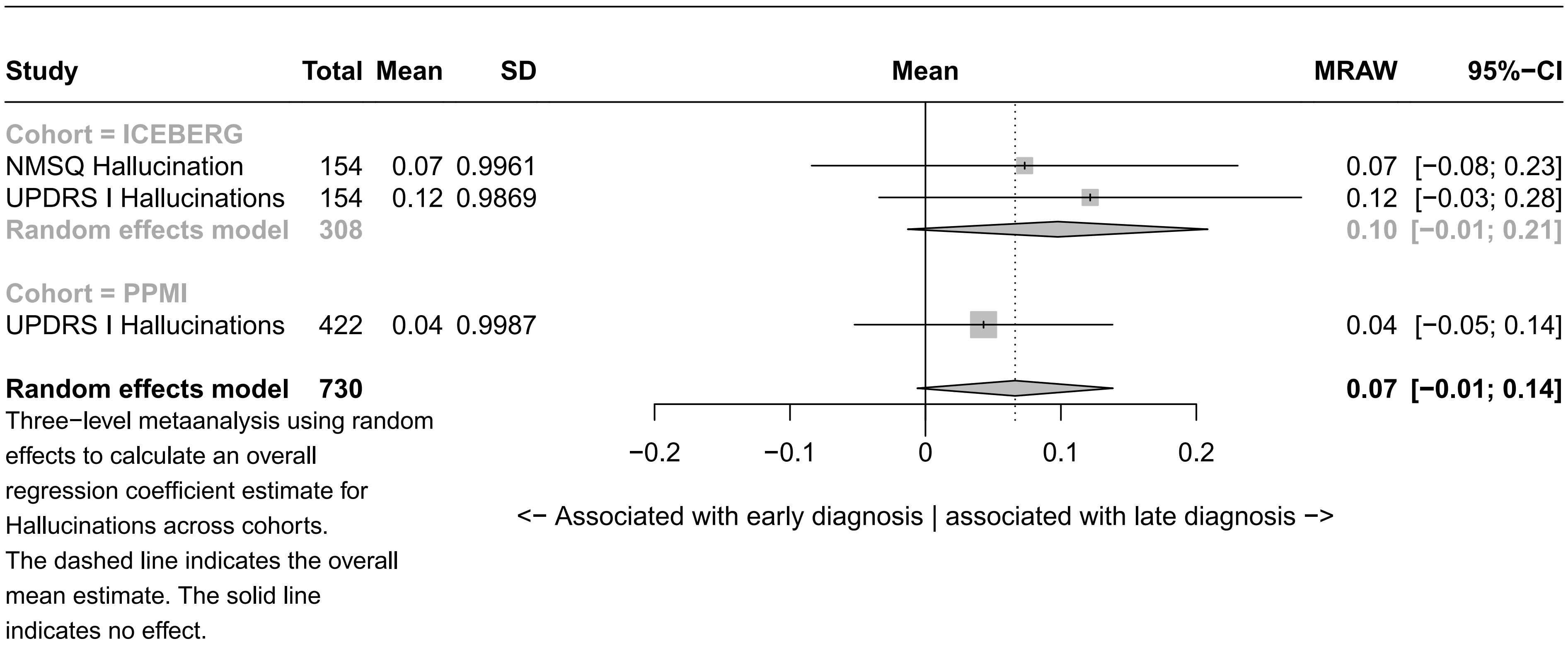

Forest plot for domain Pain

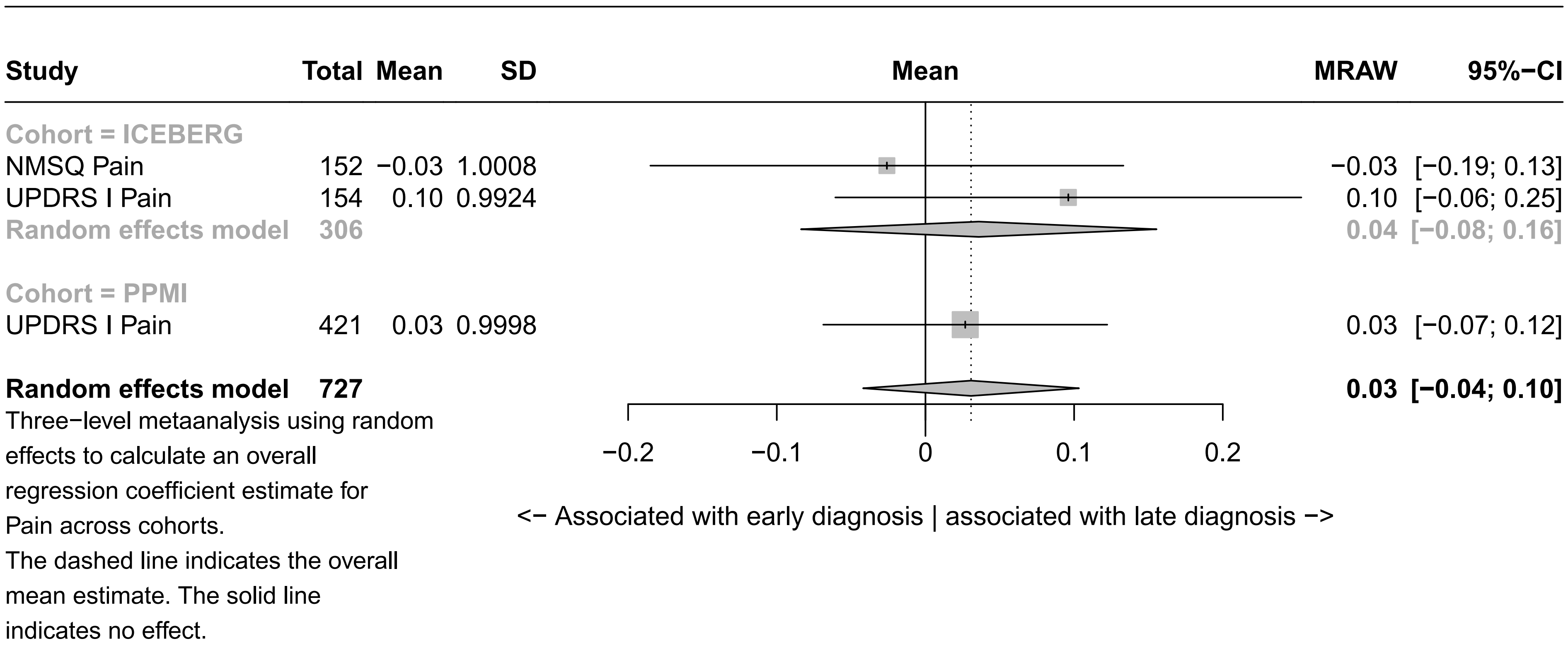

Forest plot for domain RBD

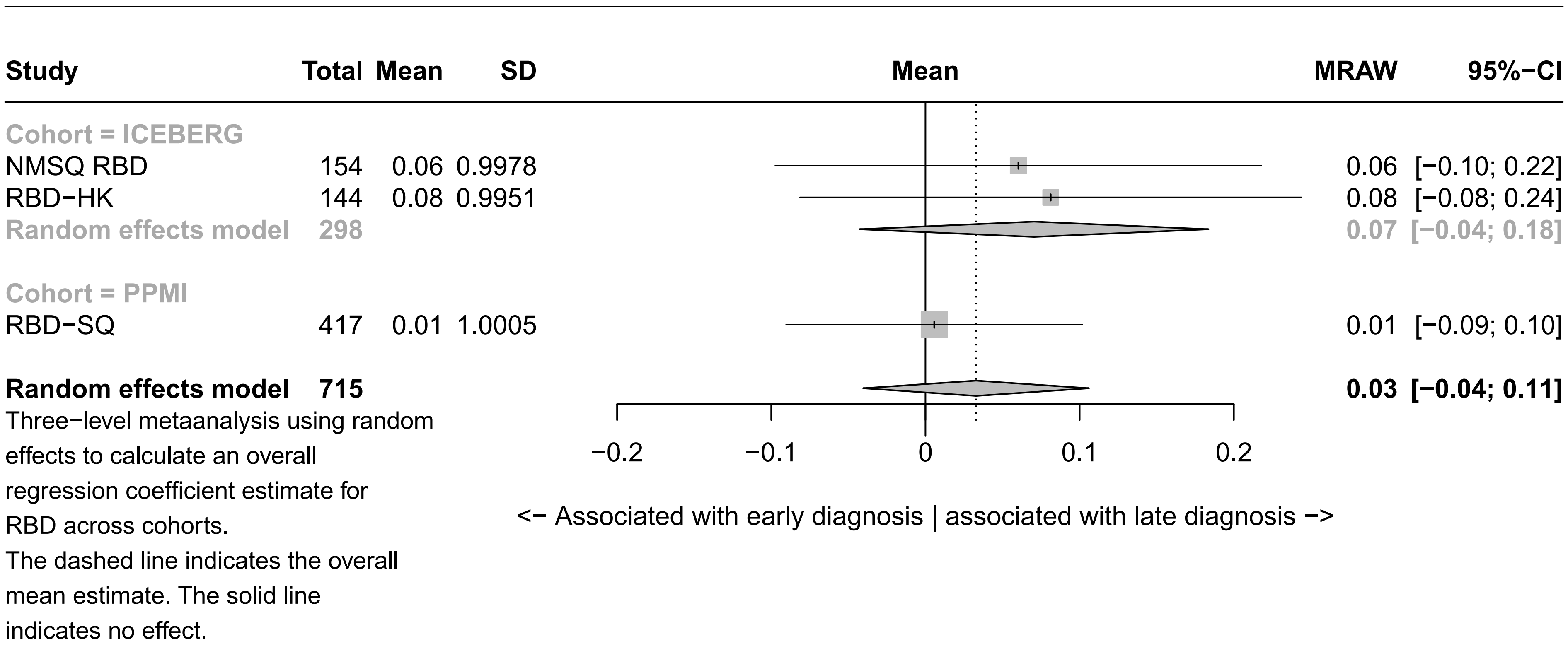

Forest plot for domain Smell

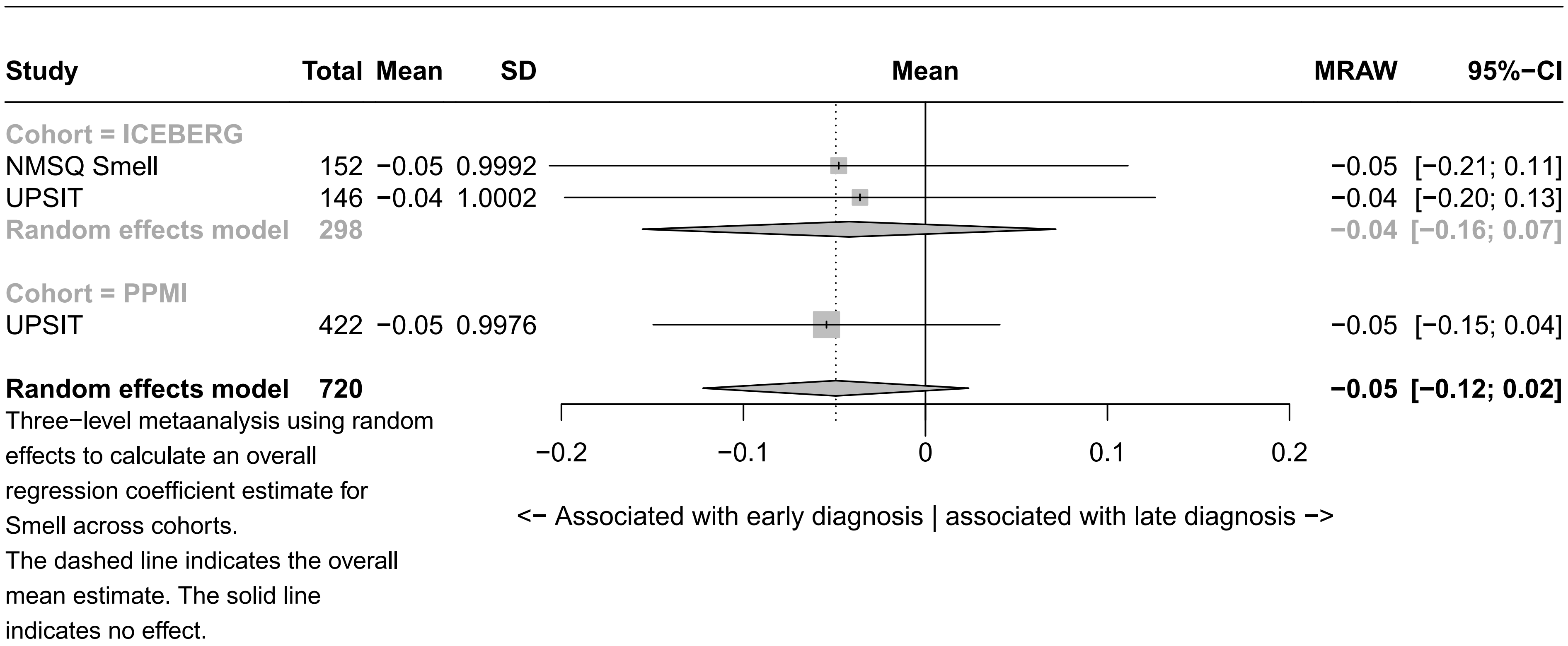

Forest plot for domain Impulsivity

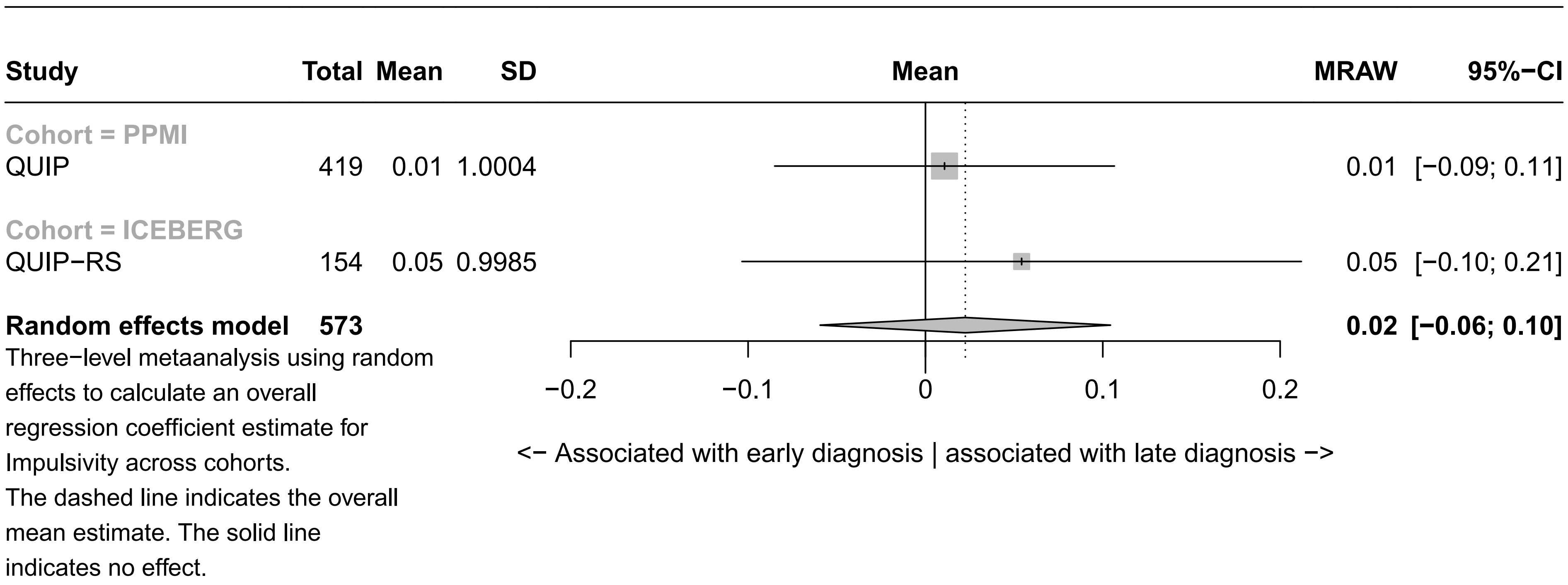

Forest plot for domain Tremor

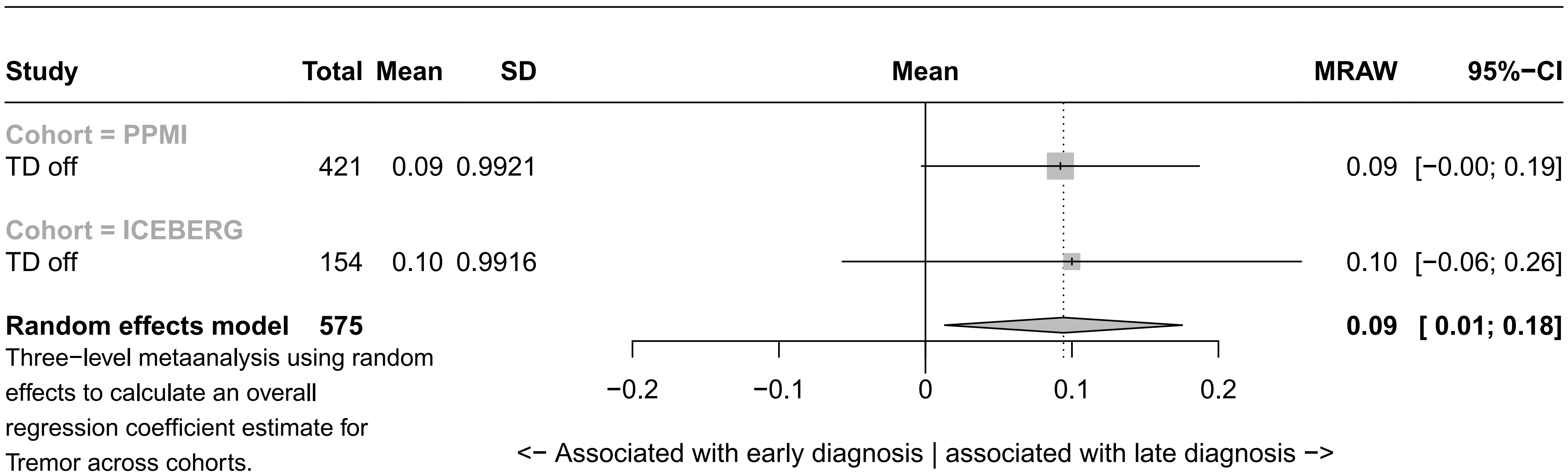

Three−level metaanalysis using random effects to calculate an overall regression coefficient estimate for Tremor across cohorts.
The dashed line indicates the overall mean estimate. The solid line indicates no effect.

Forest plot for domain Fatigue

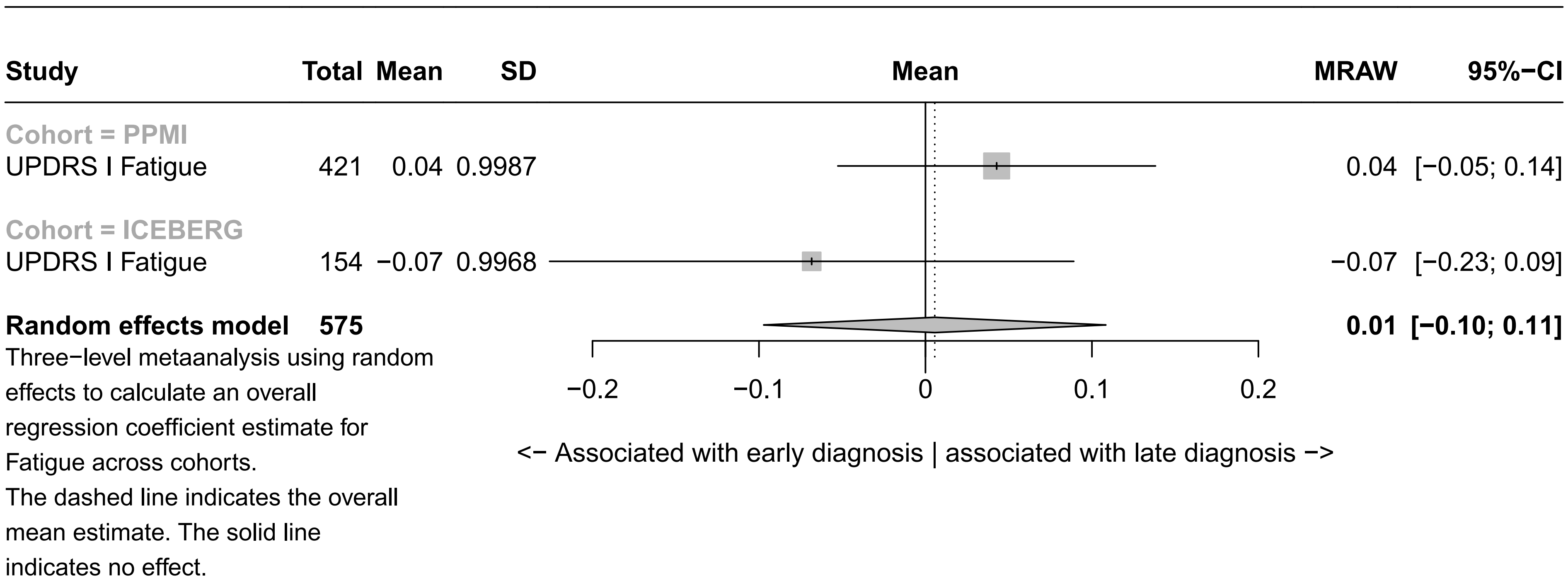

Forest plot for domain Motor symptoms

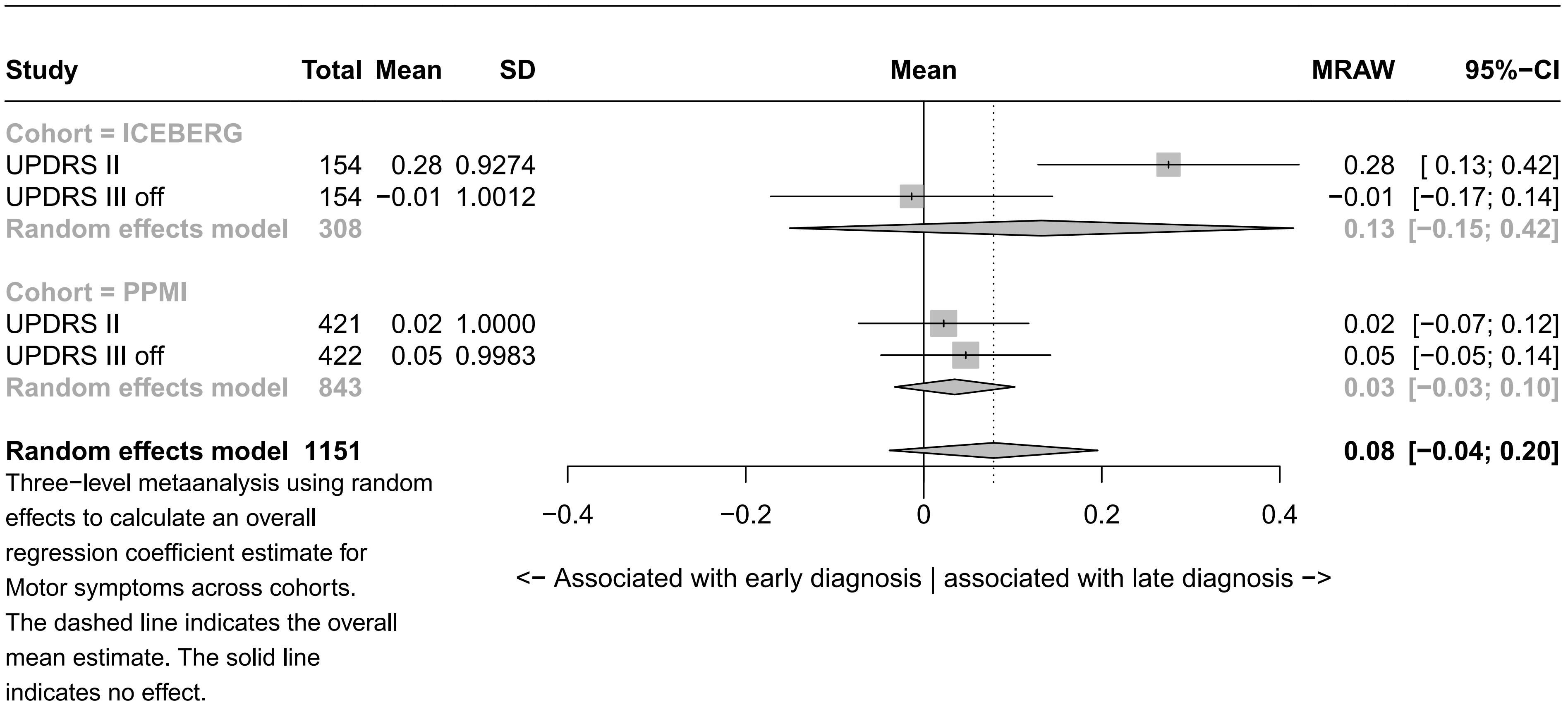

Forest plots for correlation of initial symptom domains with model-derived time shifts

Forest plot for domain Axial & PIGD

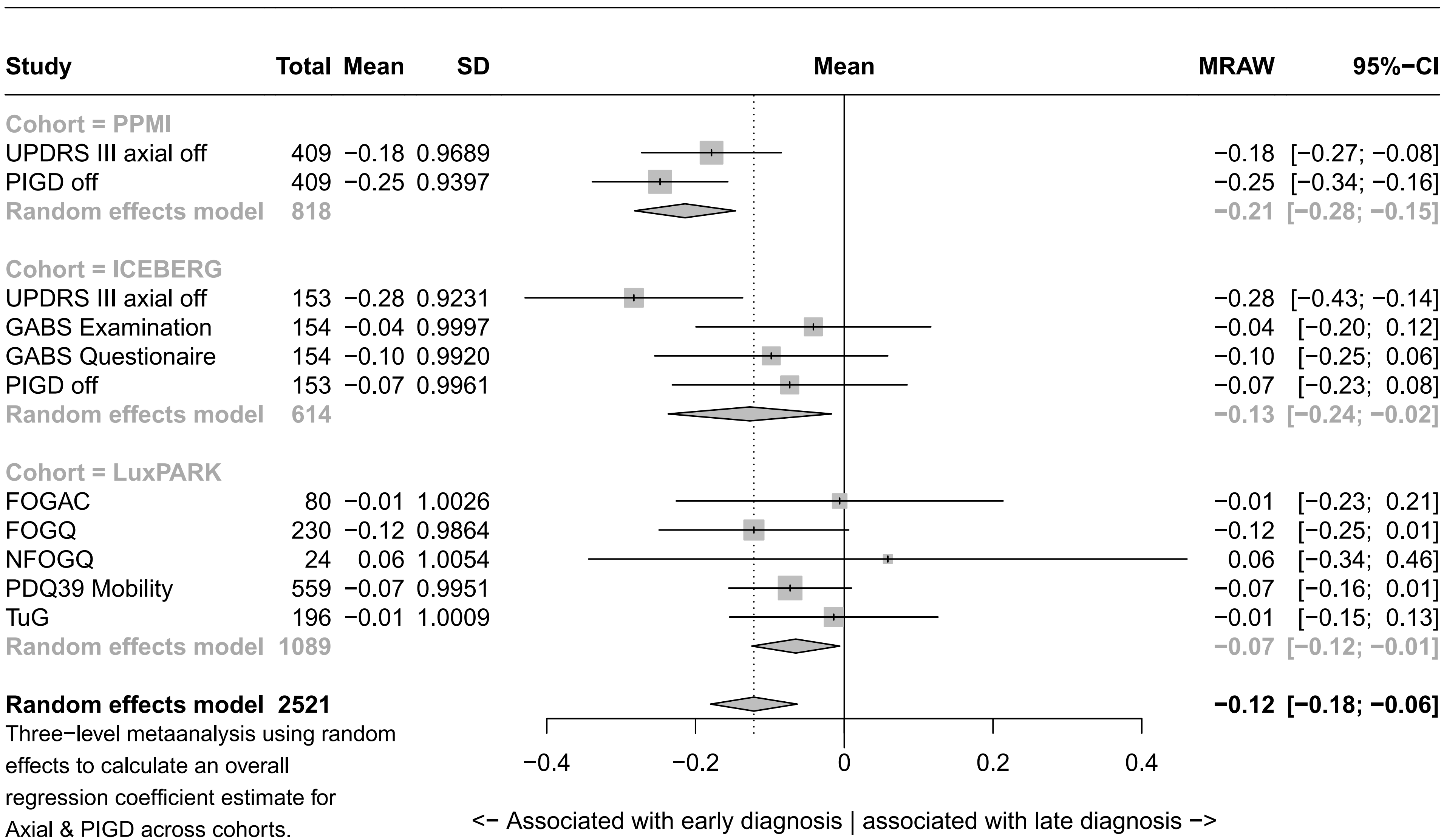

Three−level metaanalysis using random effects to calculate an overall regression coefficient estimate for Axial & PIGD across cohorts. The dashed line indicates the overall mean estimate. The solid line indicates no effect.

Forest plot for domain Depression

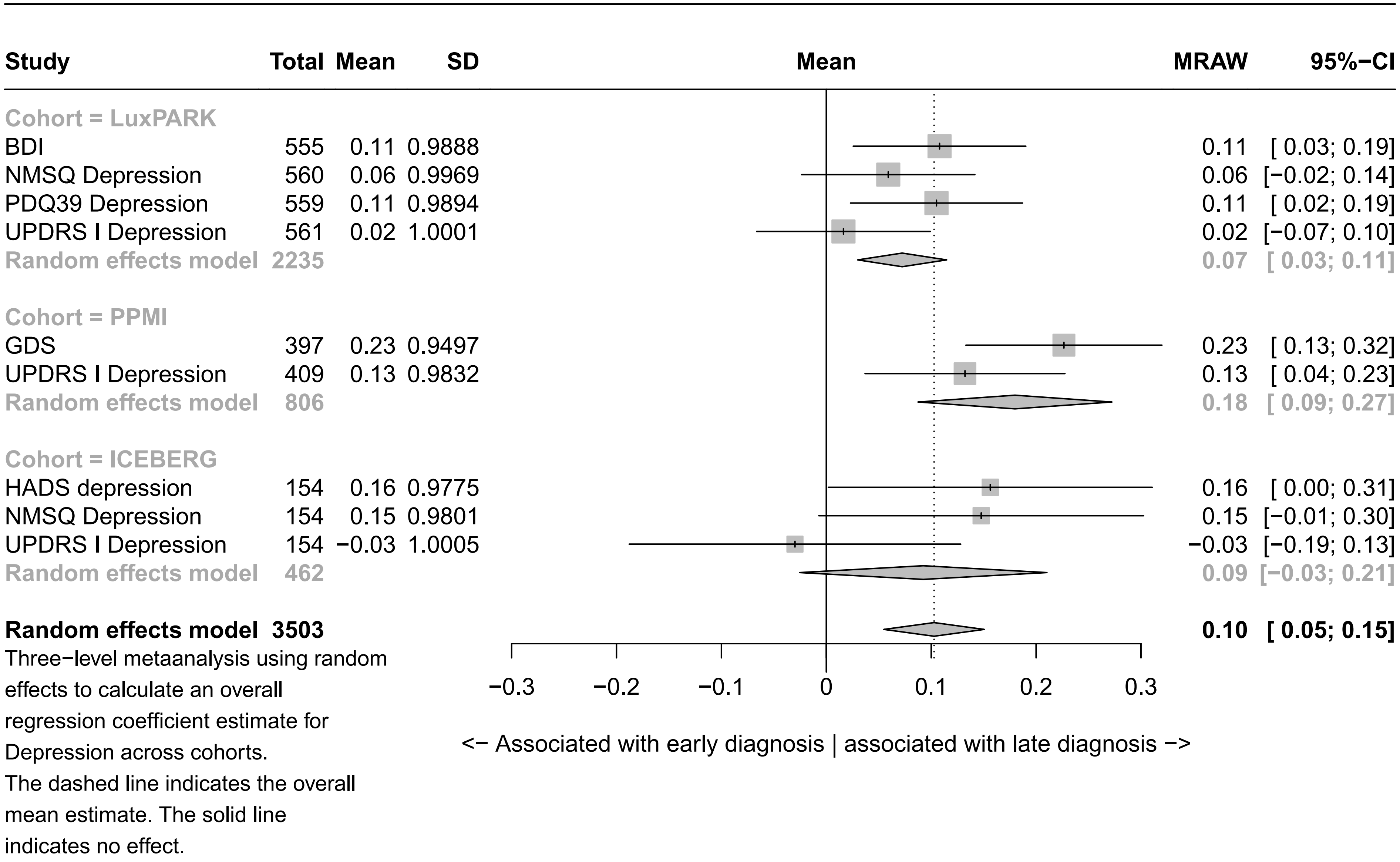

Forest plot for domain Overall severity

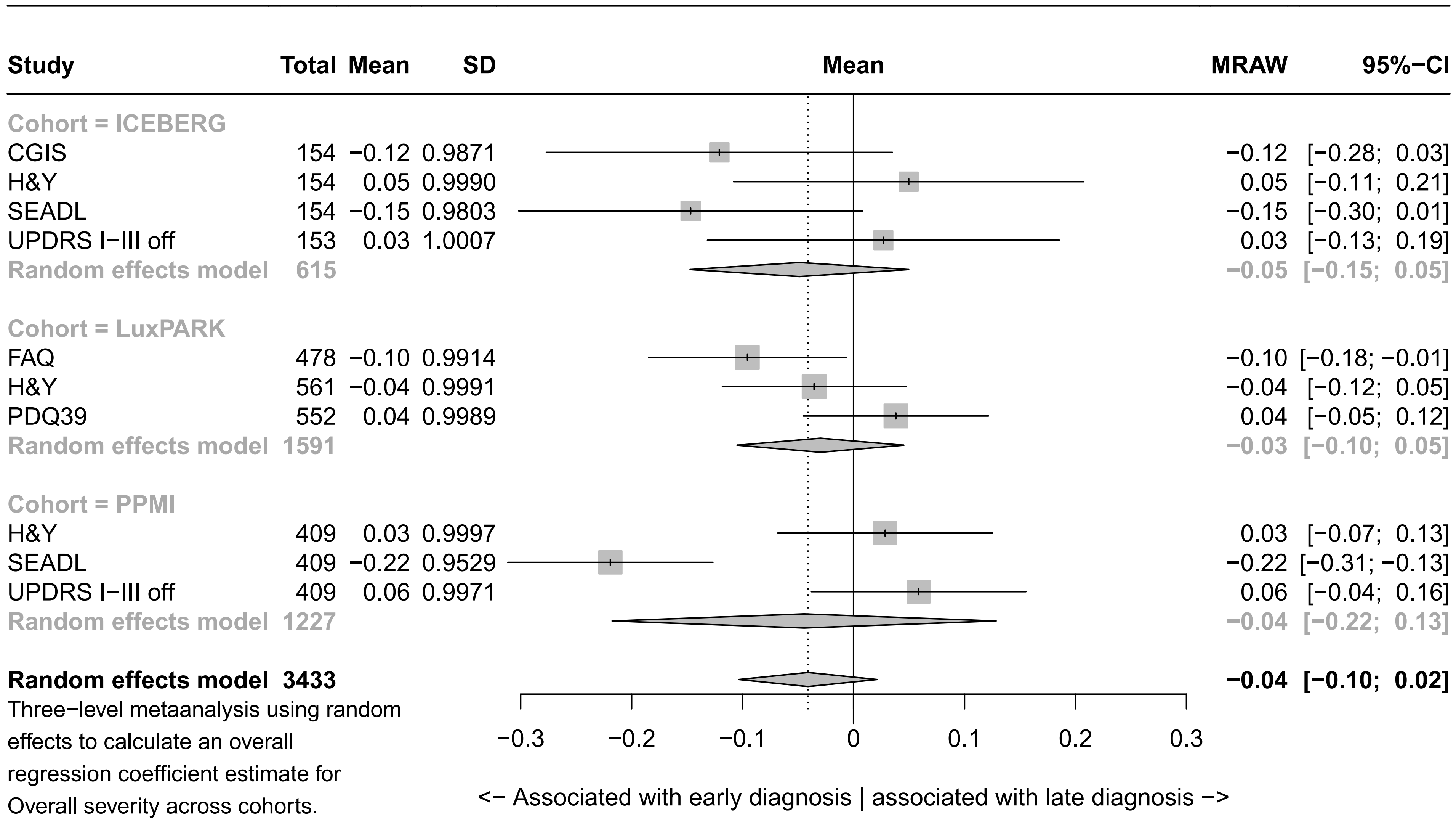

Three−level metaanalysis using random effects to calculate an overall regression coefficient estimate for Overall severity across cohorts. The dashed line indicates the overall mean estimate. The solid line indicates no effect.

Forest plot for domain Apathy

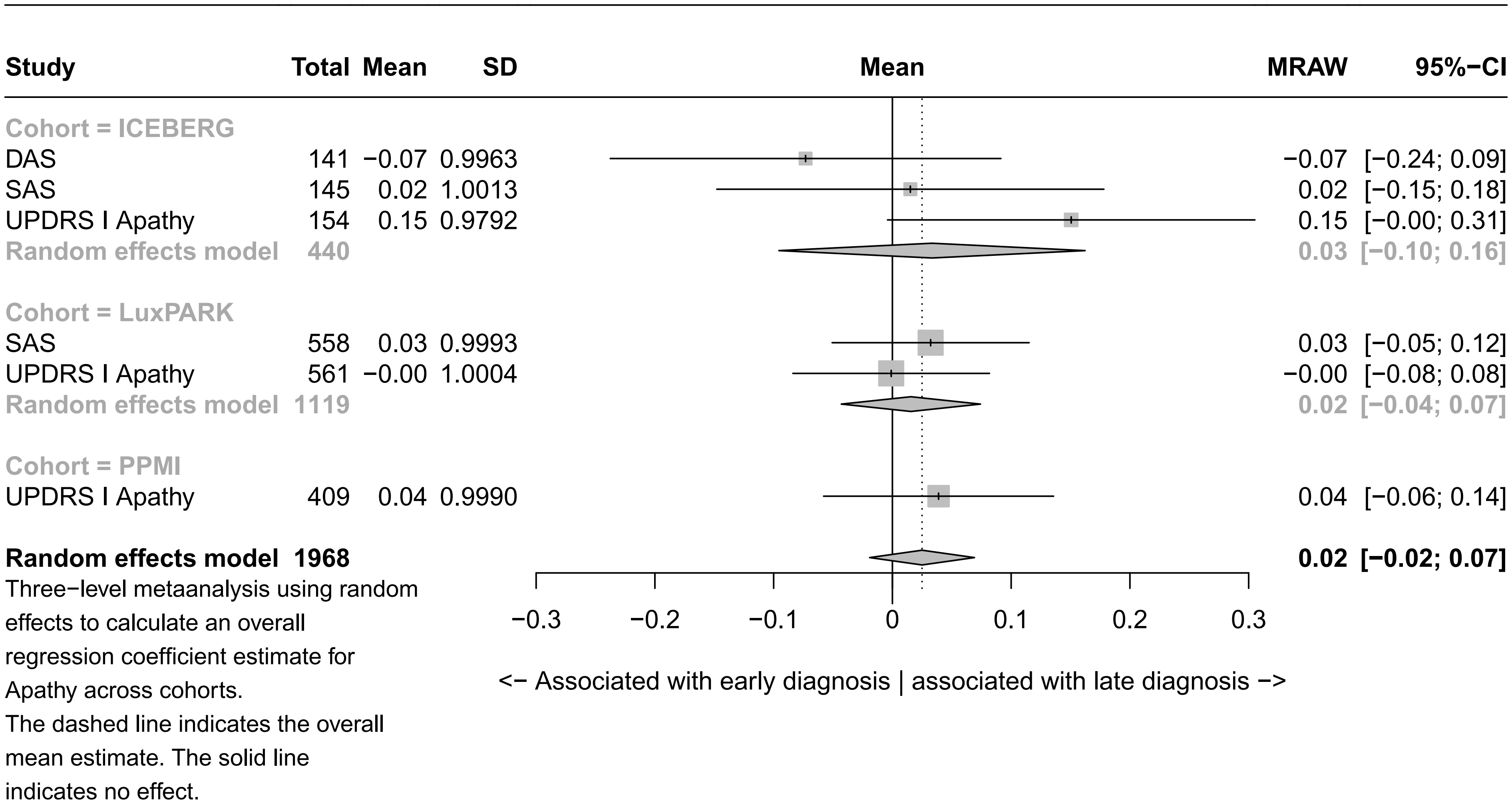

Forest plot for domain Sleep

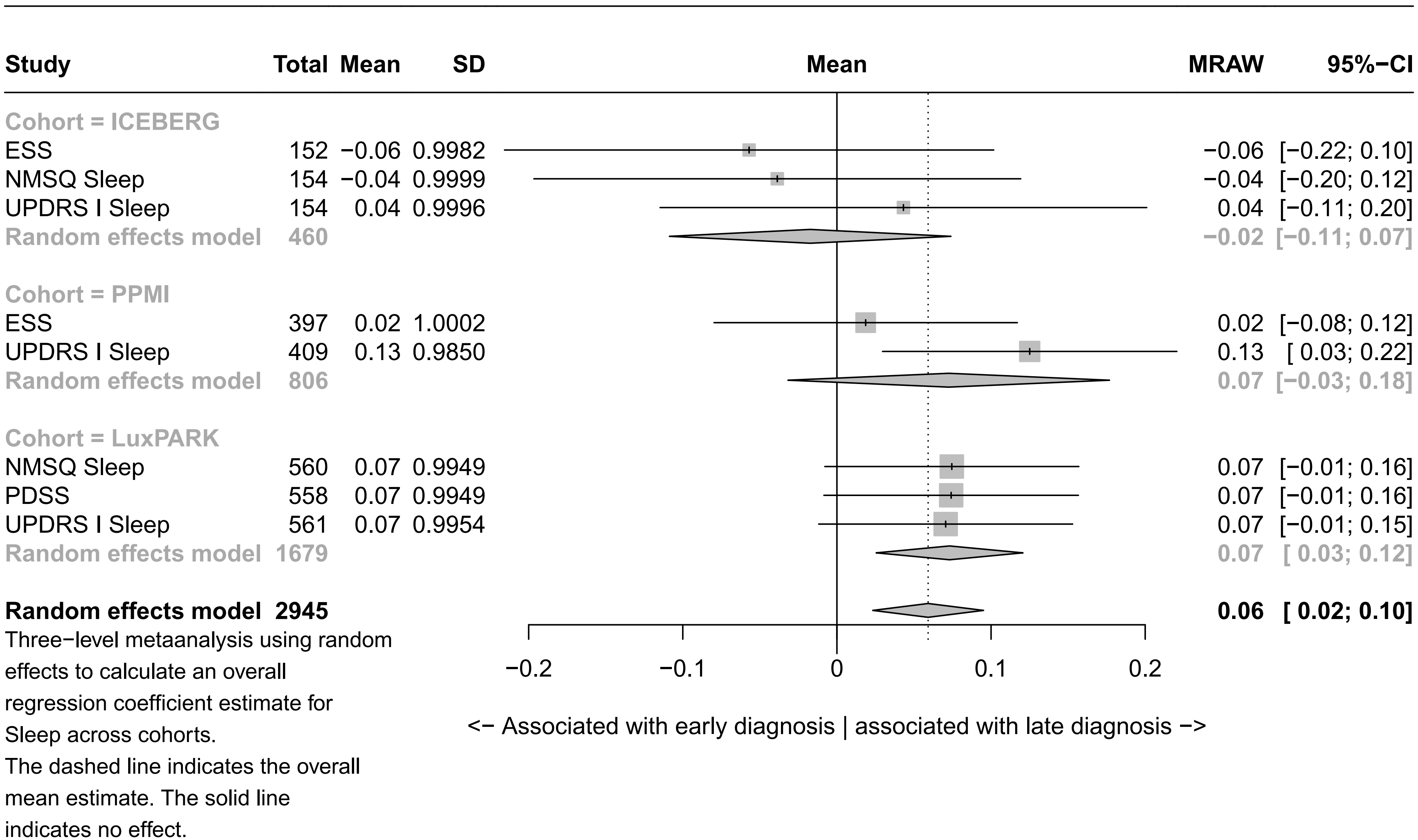

Forest plot for domain Overall cognition

Study	Total	Mean	SD	MRAW	95%−CI
**Cohort = LuxPARK**					
FAB	57	0.06	1.0000	0.06	[−0.20; 0.32]
MoCA	561	−0.10	0.9908	−0.10	[−0.18; −0.02]
NMSQ Cognition	559	0.05	0.9979	0.05	[−0.03; 0.13]
PDQ39 Cognition	560	0.10	0.9906	0.10	[0.02; 0.18]
SIQCDE	473	−0.05	0.9981	−0.05	[−0.14; 0.04]
UPDRS I Cognition	561	−0.01	1.0003	−0.01	[−0.09; 0.08]
**Random effects model**	**2771**			**0.00**	**[−0.06; 0.07]**
**Cohort = ICEBERG**					
FAB	145	−0.08	0.9957	−0.08	[−0.24; 0.08]
MATTIS	145	−0.09	0.9942	−0.09	[−0.25; 0.08]
MoCA	154	−0.07	0.9964	−0.07	[−0.23; 0.09]
NMSQ Cognition	154	0.09	0.9932	0.09	[−0.07; 0.25]
UPDRS I Cognition	154	0.12	0.9883	0.12	[−0.04; 0.27]
**Random effects model**	**752**			**−0.00**	**[−0.09; 0.08]**
**Cohort = PPMI**					
MoCA	391	0.07	0.9958	0.07	[−0.03; 0.17]
UPDRS I Cognition	409	−0.03	0.9993	−0.03	[−0.13; 0.06]
**Random effects model**	**800**			**0.02**	**[−0.08; 0.12]**
**Random effects model**	**4323**			**0.00**	**[−0.04; 0.05]**

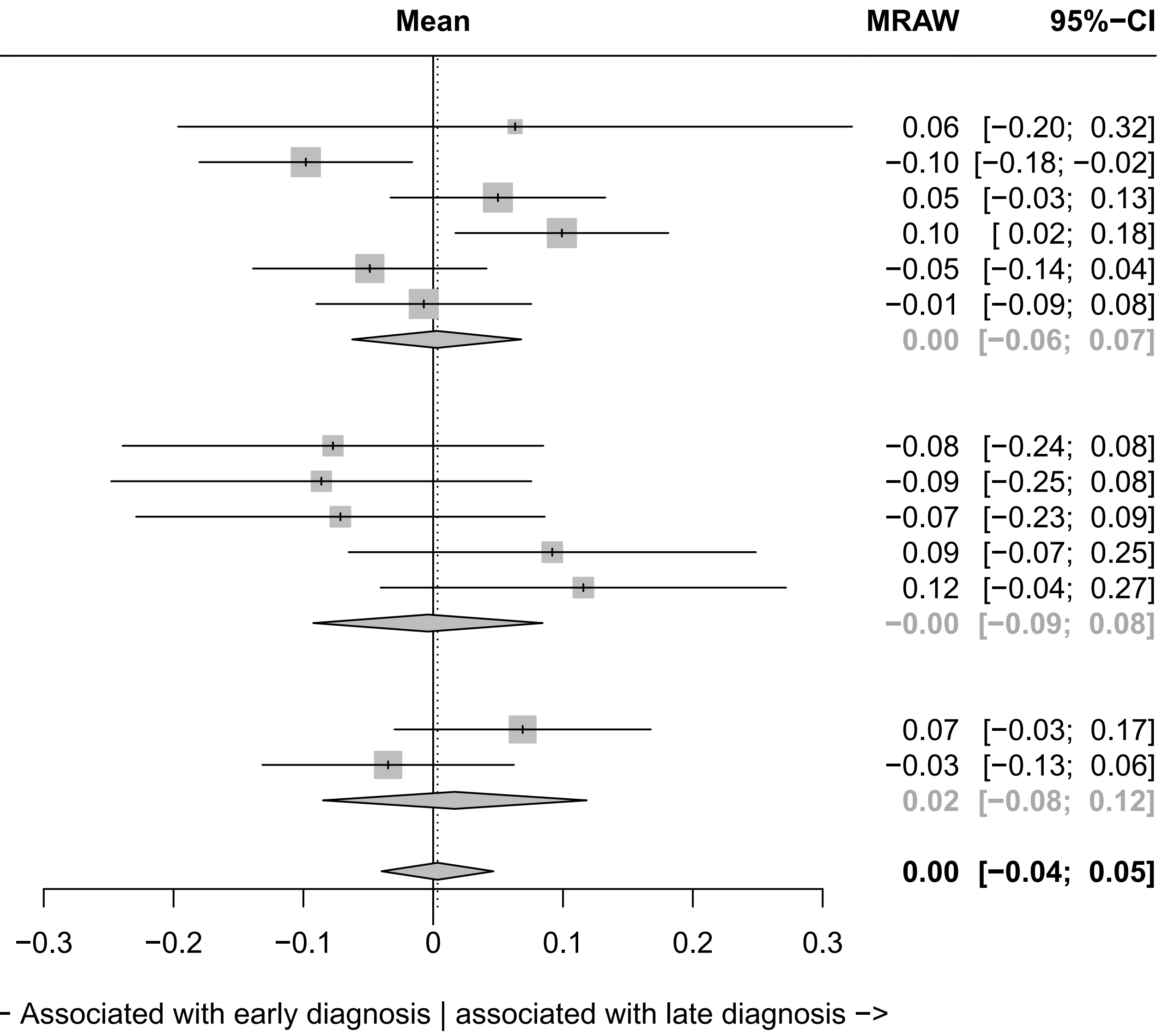

Three−level metaanalysis using random effects to calculate an overall regression coefficient estimate for Overall cognition across cohorts. The dashed line indicates the overall mean estimate. The solid line indicates no effect.

Forest plot for domain Anxiety

Study	Total	Mean	SD	MRAW	95%−CI
**Cohort = ICEBERG**					
HADS anxiety	154	0.26	0.9349	0.26	[0.11; 0.41]
NMSQ Anxiety	153	0.05	0.9992	0.05	[−0.11; 0.21]
UPDRS I Anxiety	154	0.12	0.9882	0.12	[−0.04; 0.27]
**Random effects model**	**461**			**0.14**	**[0.02; 0.27]**
**Cohort = LuxPARK**					
NMSQ Anxiety	559	0.01	1.0003	0.01	[−0.07; 0.09]
PDQ39 Anxiety	561	0.08	0.9939	0.08	[−0.00; 0.16]
UPDRS I Anxiety	561	0.03	0.9994	0.03	[−0.05; 0.11]
**Random effects model**	**1681**			**0.04**	**[−0.01; 0.09]**
**Cohort = PPMI**					
STA	395	0.30	0.9120	0.30	[0.21; 0.39]
UPDRS I Anxiety	409	0.18	0.9694	0.18	[0.08; 0.27]
**Random effects model**	**804**			**0.24**	**[0.12; 0.36]**
**Random effects model**	**2946**			**0.13**	**[0.05; 0.20]**

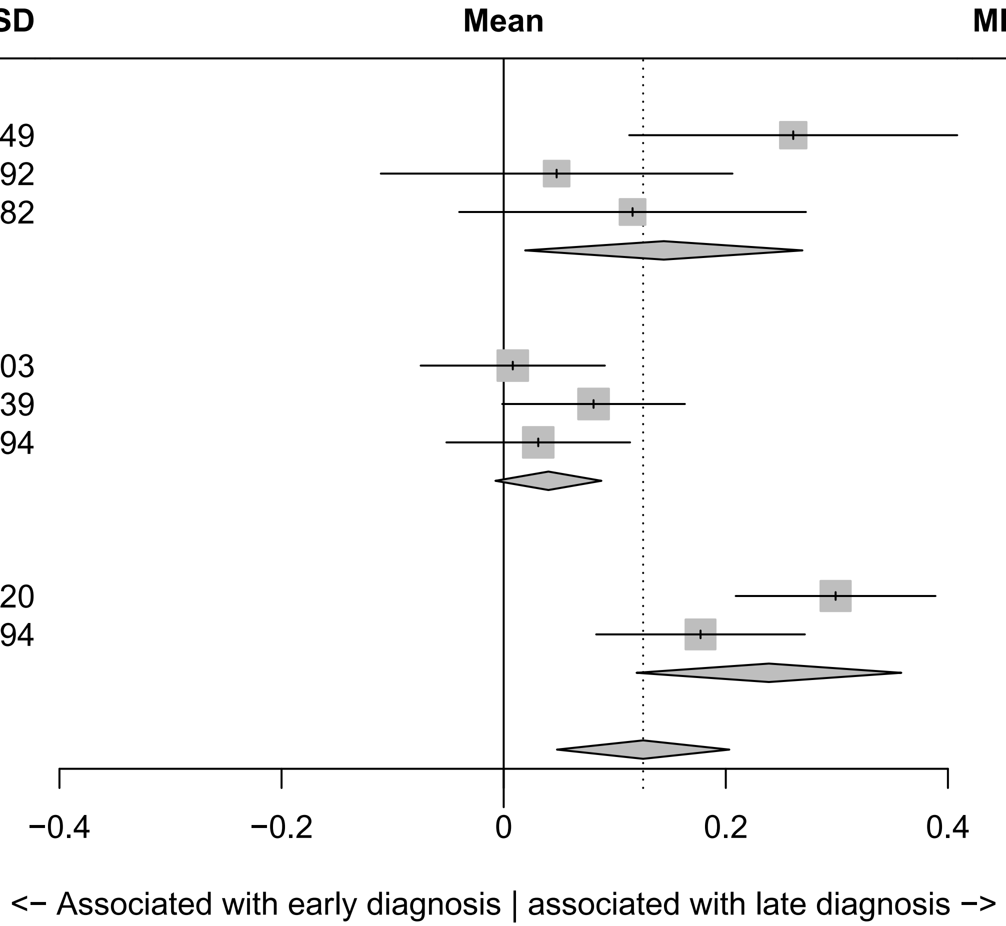

Three−level metaanalysis using random effects to calculate an overall regression coefficient estimate for Anxiety across cohorts.
The dashed line indicates the overall mean estimate. The solid line indicates no effect.

Forest plot for domain Non motor symptoms

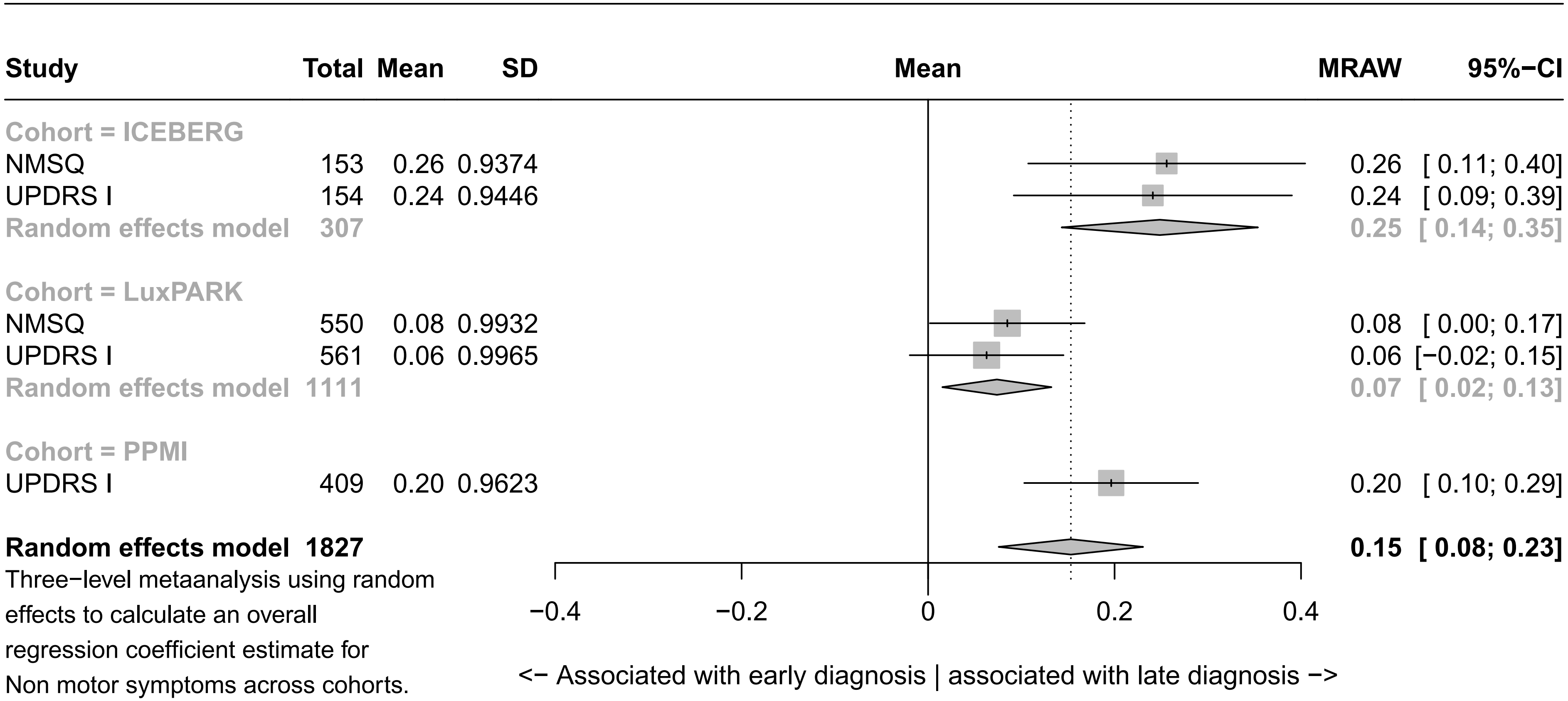

Three−level metaanalysis using random effects to calculate an overall regression coefficient estimate for Non motor symptoms across cohorts. The dashed line indicates the overall mean estimate. The solid line indicates no effect.

Forest plot for domain Autonomic

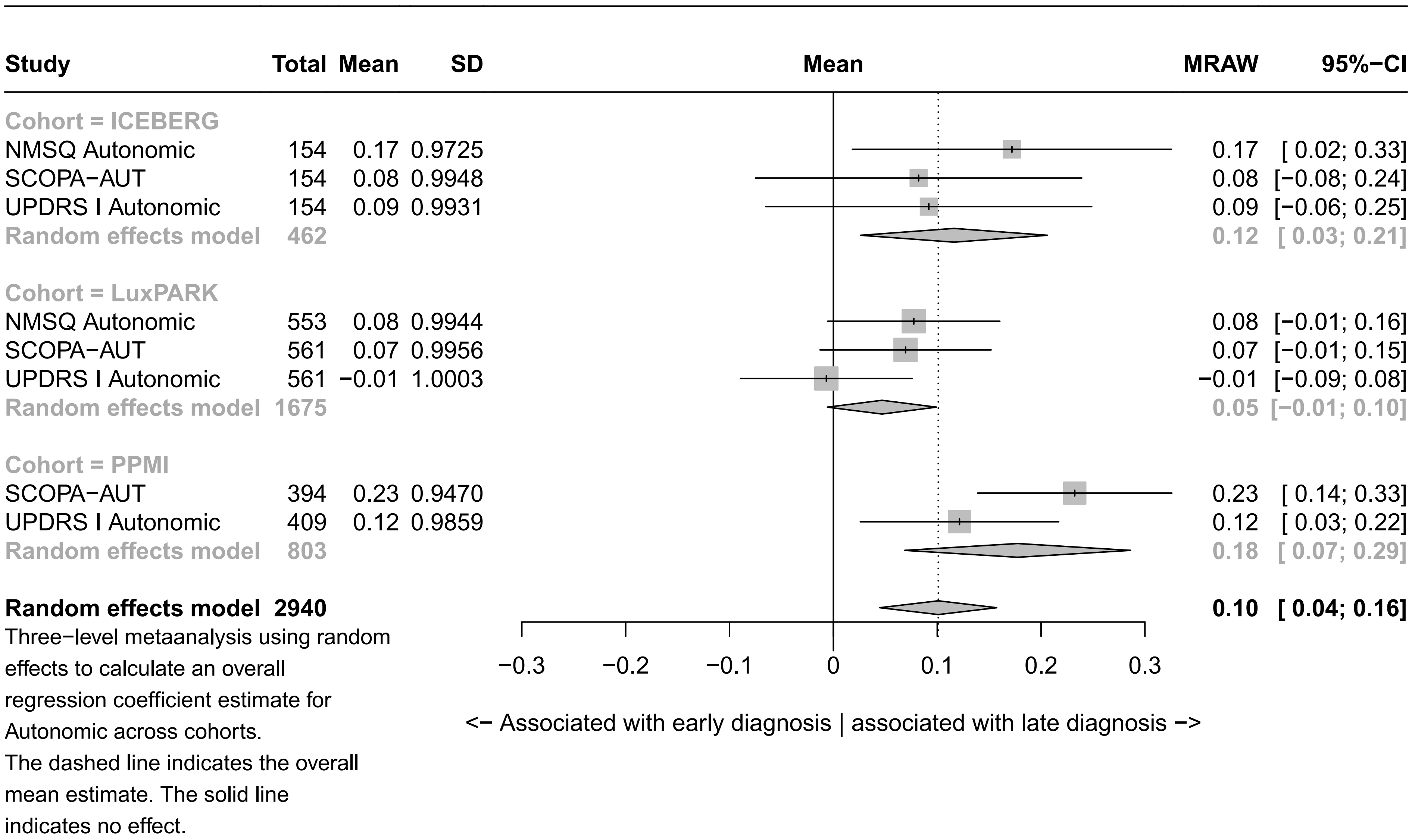

Forest plot for domain Hallucinations

Study	Total	Mean	SD	MRAW	95%−CI
Cohort = LuxPARK					
NMSQ Hallucination	560	−0.06	0.9968	−0.06	[−0.14; 0.02]
UPDRS I Hallucinations	561	−0.09	0.9924	−0.09	[−0.17; −0.01]
Random effects model	1121			−0.07	[−0.13; −0.02]
Cohort = ICEBERG					
NMSQ Hallucination	154	0.13	0.9845	0.13	[−0.02; 0.29]
UPDRS I Hallucinations	154	−0.03	1.0005	−0.03	[−0.19; 0.13]
Random effects model	308			0.05	[−0.11; 0.21]
Cohort = PPMI					
UPDRS I Hallucinations	409	−0.05	0.9978	−0.05	[−0.15; 0.04]
**Random effects model**	**1838**			**−0.05**	**[−0.09; −0.00]**

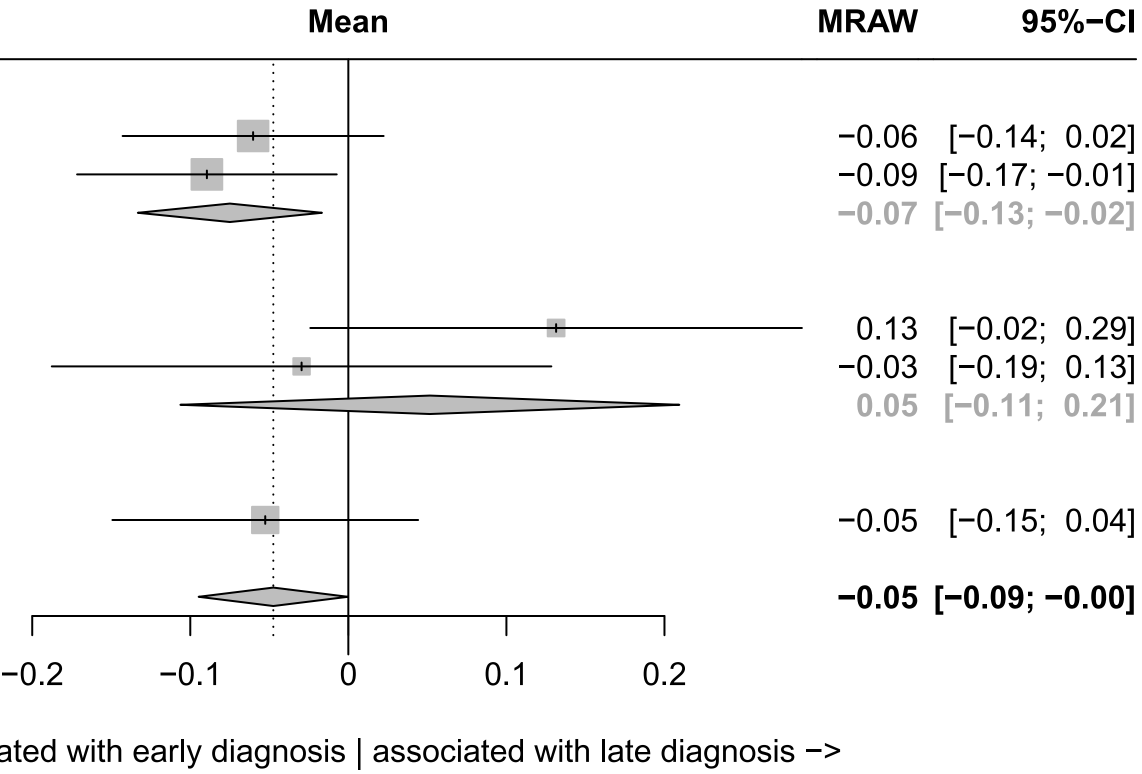

Three−level metaanalysis using random effects to calculate an overall regression coefficient estimate for Hallucinations across cohorts. The dashed line indicates the overall mean estimate. The solid line indicates no effect.

Forest plot for domain Pain

Study	Total	Mean	SD	MRAW	95%−CI
Cohort = LuxPARK					
NMSQ Pain	560	0.03	0.9997	0.03	[−0.06; 0.11]
PDQ39 Pain	560	0.12	0.9865	0.12	[0.04; 0.20]
UPDRS I Pain	561	0.07	0.9959	0.07	[−0.02; 0.15]
Random effects model	1681			0.07	[0.02; 0.12]
Cohort = ICEBERG					
NMSQ Pain	153	0.00	1.0014	0.00	[−0.16; 0.16]
UPDRS I Pain	154	0.05	0.9989	0.05	[−0.11; 0.21]
Random effects model	307			0.03	[−0.08; 0.14]
Cohort = PPMI					
UPDRS I Pain	409	0.19	0.9634	0.19	[0.10; 0.29]
**Random effects model**	**2397**			**0.08**	**[0.03; 0.14]**

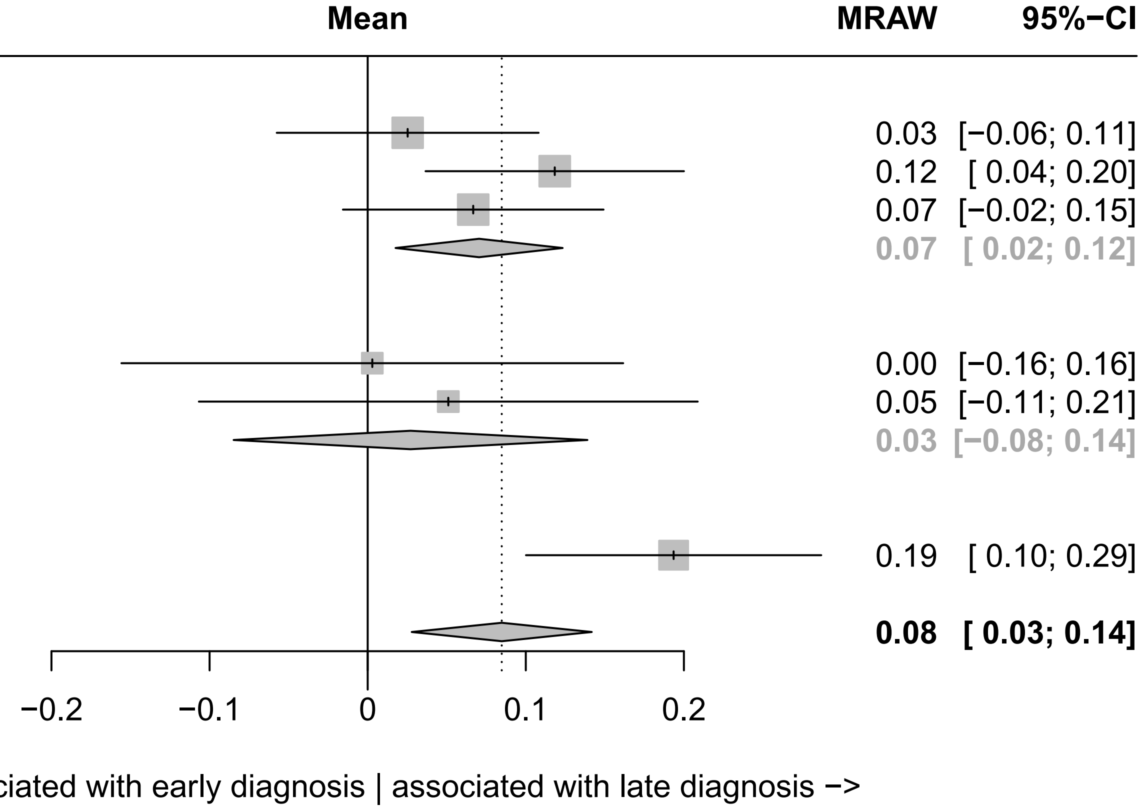

Three−level metaanalysis using random effects to calculate an overall regression coefficient estimate for Pain across cohorts.
The dashed line indicates the overall mean estimate. The solid line indicates no effect.

Forest plot for domain RBD

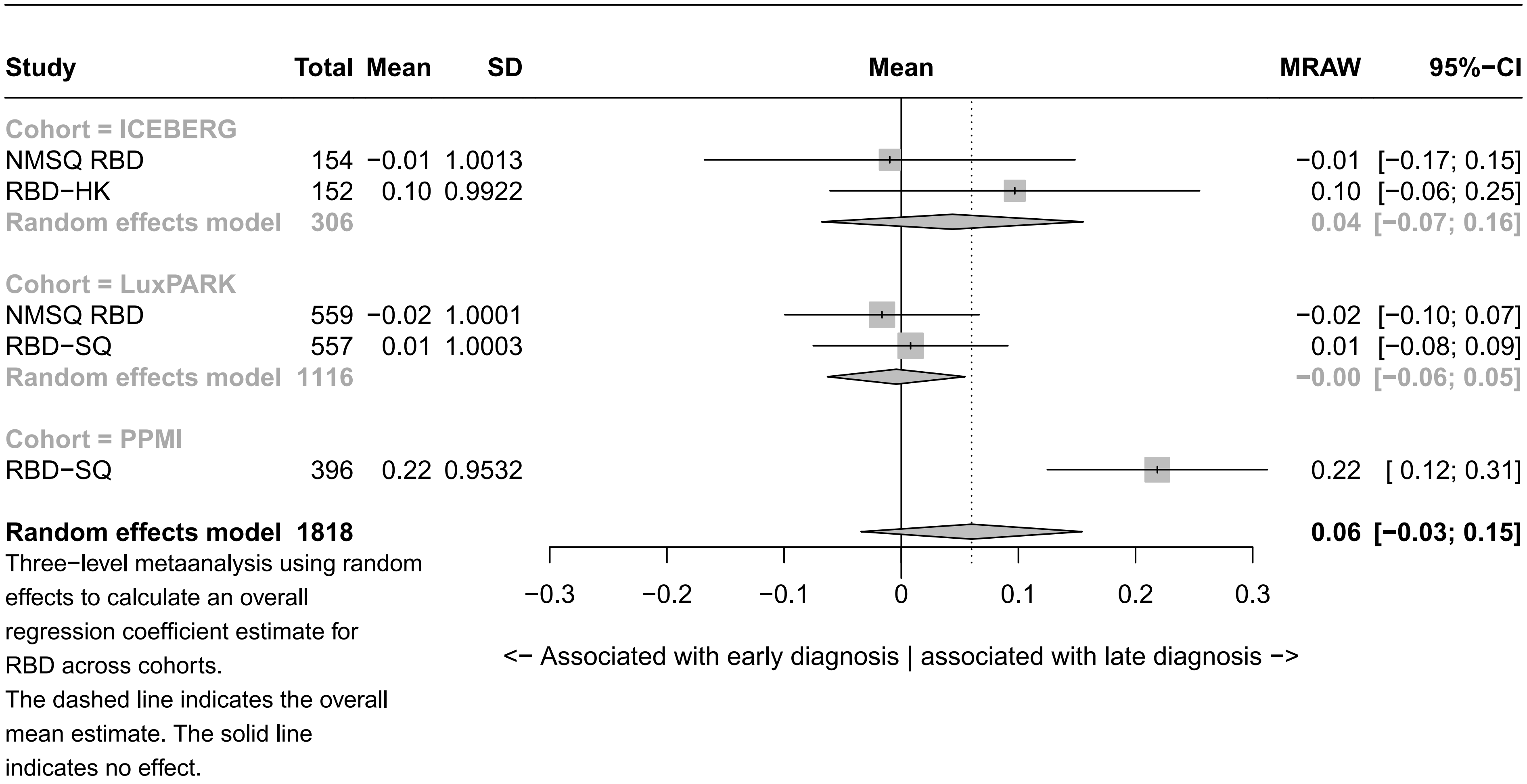

Forest plot for domain Smell

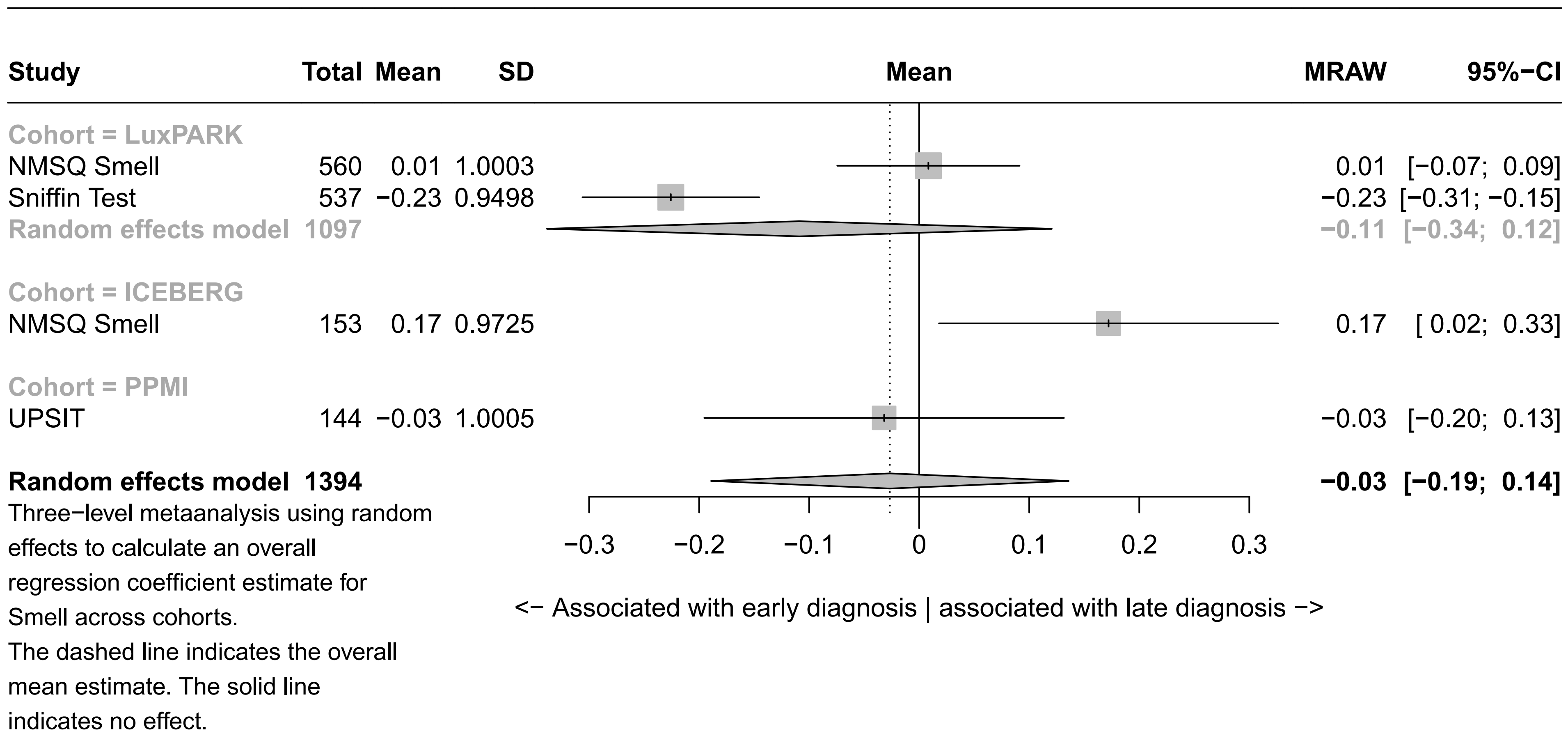

Forest plot for domain Motor symptoms

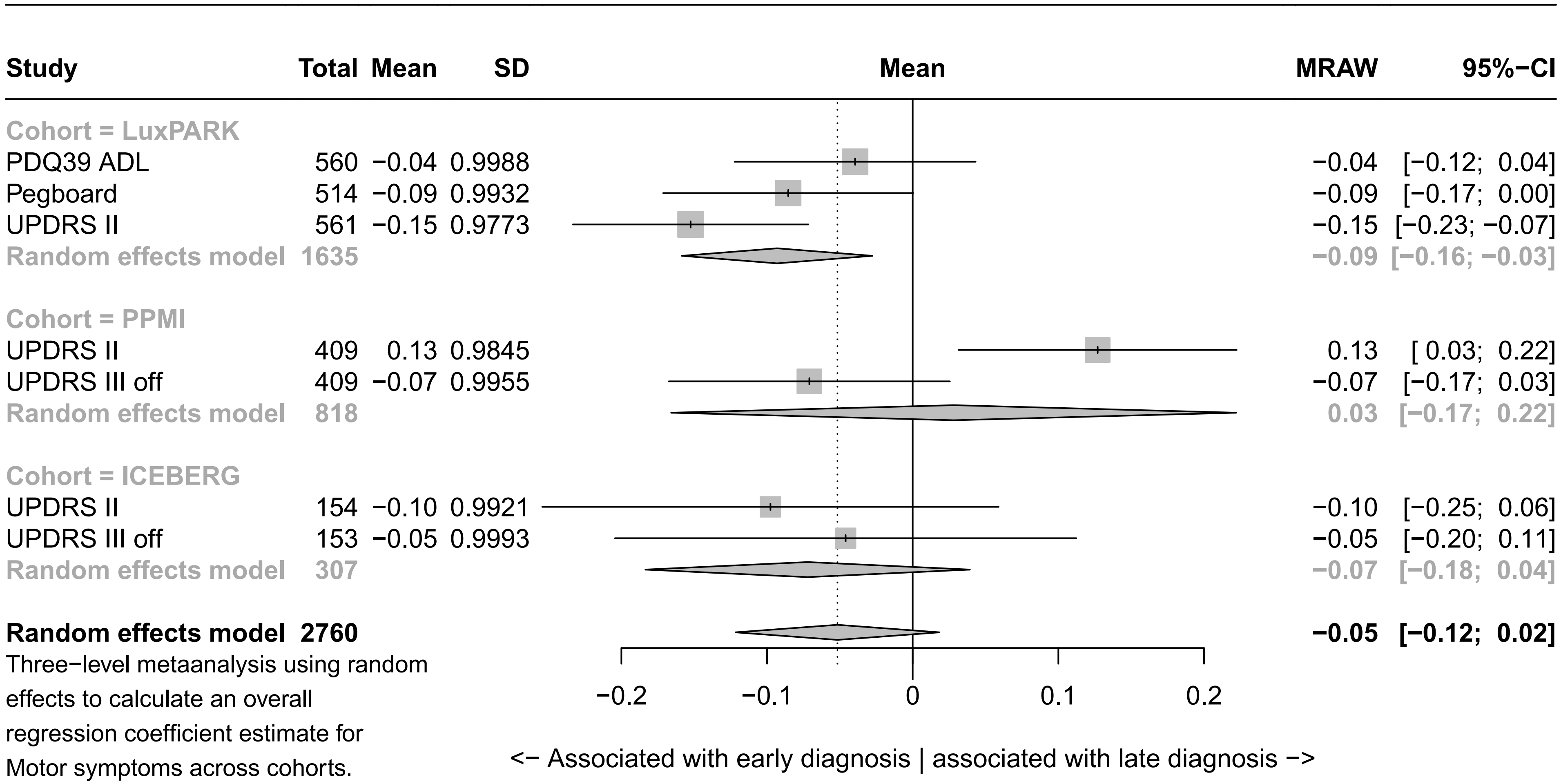

Three−level metaanalysis using random effects to calculate an overall regression coefficient estimate for Motor symptoms across cohorts. The dashed line indicates the overall mean estimate. The solid line indicates no effect.

Forest plot for domain Impulsivity

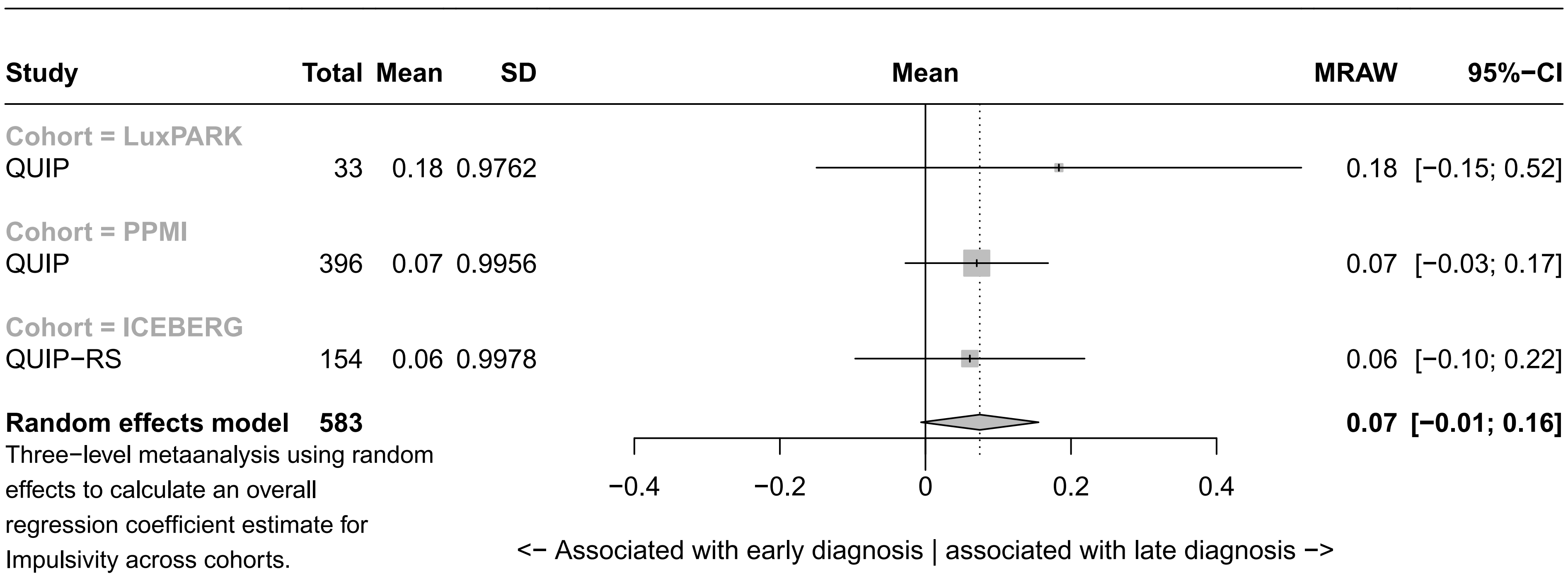

Three−level metaanalysis using random effects to calculate an overall regression coefficient estimate for Impulsivity across cohorts.
The dashed line indicates the overall mean estimate. The solid line indicates no effect.

Forest plot for domain Tremor

Study	Total	Mean	SD	MRAW	95%−CI
Cohort = ICEBERG					
TD off	153	0.05	0.9985	0.05	[−0.10; 0.21]
Cohort = PPMI					
TD off	409	−0.08	0.9942	−0.08	[−0.18; 0.02]
**Random effects model**	**562**			**−0.03**	**[−0.16; 0.10]**

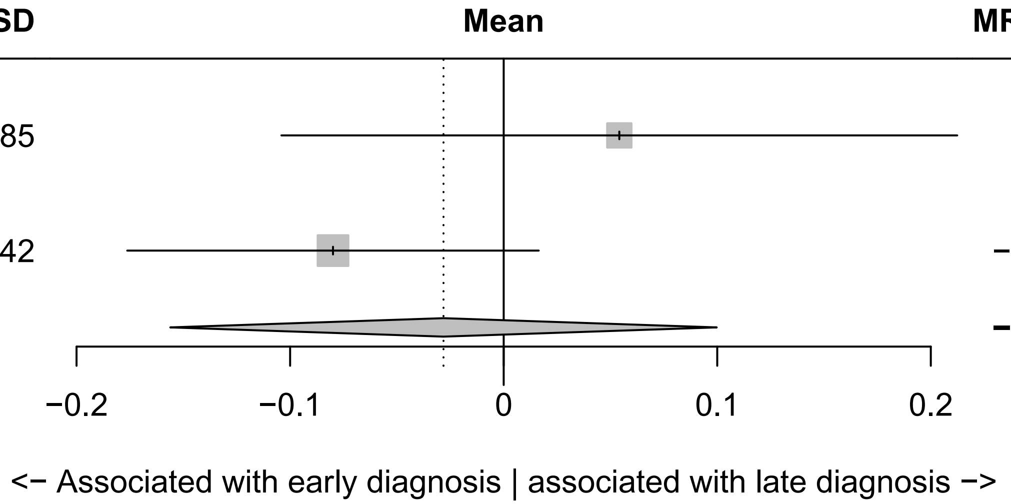

Three−level metaanalysis using random effects to calculate an overall regression coefficient estimate for Tremor across cohorts.
The dashed line indicates the overall mean estimate. The solid line indicates no effect.

Forest plot for domain Fatigue

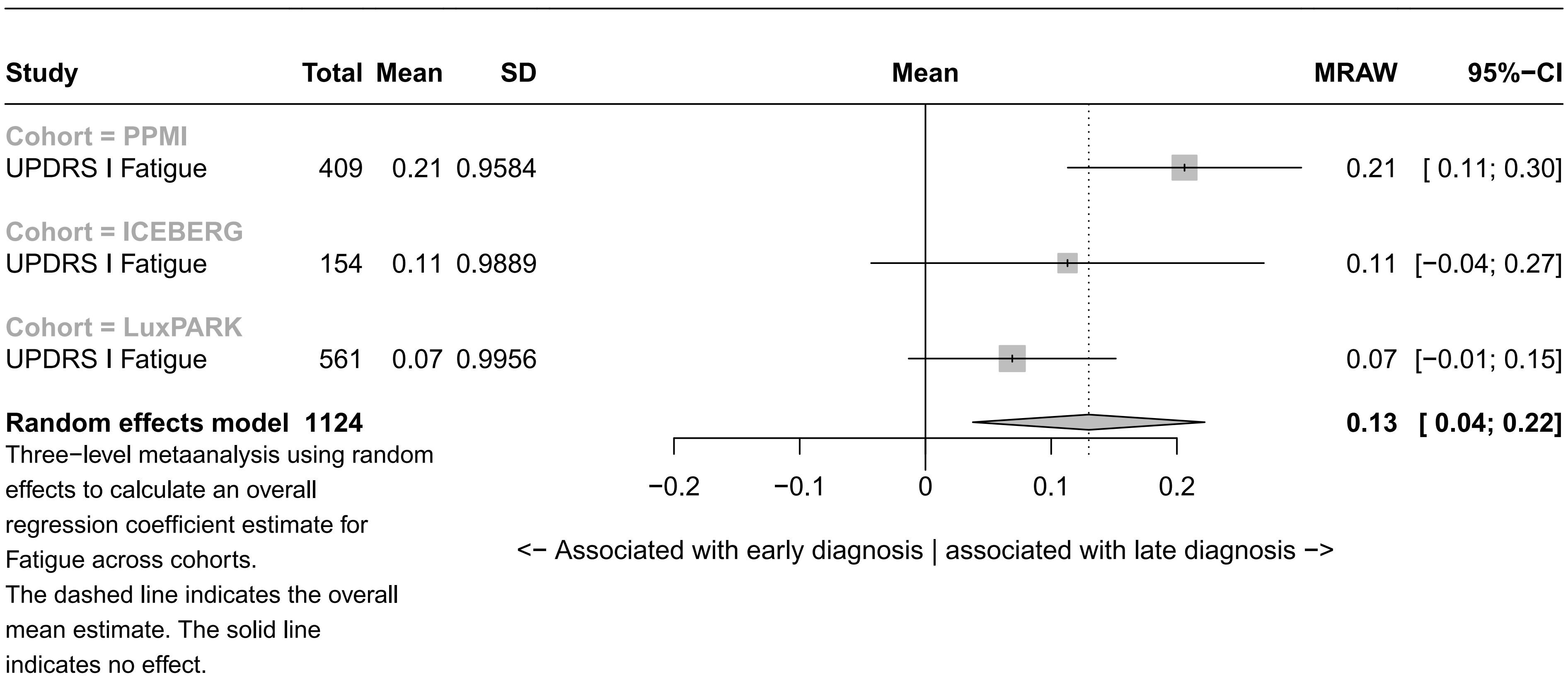

Acknowledgment

Parkinson’s Progression Markers Initiative

Data used in the preparation of this article were obtained from the Parkinson’s Progression Markers Initiative (PPMI) database www.ppmi-info.org/data. PPMI—a public-private partnership – is funded by the Michael J. Fox Foundation for Parkinson’s Research and funding partners. A list of names of all the PPMI funding partners can be found at www.ppmi-info.org/about-ppmi/who-we-are/study-sponsors/.

ICEBERG study group

Steering committee: Marie Vidailhet, MD, PhD, (Pitié-Salpêtrière Hospital, Paris, principal investigator of ICEBERG), Jean-Christophe Corvol, MD, PhD (Pitié-Salpêtrière Hospital, Paris, scientific lead), Isabelle Arnulf, MD, PhD (Pitié-Salpêtrière Hospital, Paris, member of the steering committee), Stéphane Lehericy, MD, PhD (Pitié-Salpêtrière Hospital, Paris, member of the steering committee);

Clinical data: Marie Vidailhet, MD, PhD, (Pitié-Salpêtrière Hospital, Paris, coordination), Graziella Mangone, MD, PhD (Pitié-Salpêtrière Hospital, Paris, co-coordination), Jean-Christophe Corvol, MD, PhD (Pitié-Salpêtrière Hospital, Paris), Isabelle Arnulf, MD, PhD (Pitié-Salpêtrière Hospital, Paris), Sara Sambin, MD (Pitié-Salpêtrière Hospital, Paris), Poornima Menon, MD (Pitié-Salpêtrière Hospital, Paris, Jonas Ihle, MD (Pitié-Salpêtrière Hospital, Paris), Caroline Weill, MD, (Pitié-Salpêtrière Hospital, Paris), David Grabli, MD, PhD (Pitié-Salpêtrière Hospital, Paris); Florence Cormier-Dequaire, MD (Pitié-Salpêtrière Hospital, Paris); Louise Laure Mariani, MD, PhD (Pitié-Salpêtrière Hospital, Paris), Bertrand Degos, MD, PhD (Avicenne Hospital, Bobigny);

Neuropsychological data: Richard Levy, MD (Pitié-Salpêtrière Hospital, Paris, coordination), Fanny Pineau, MS (Pitié-Salpêtrière Hospital, Paris, neuropsychologist), Julie Socha, MS (Pitié-Salpêtrière Hospital, Paris, neuropsychologist), Eve Benchetrit, MS (La Timone Hospital, Marseille, neuropsychologist), Virginie Czernecki, MS (Pitié-Salpêtrière Hospital, Paris, neuropsychologist), Marie-Alexandrine, MS (Pitié-Salpêtrière Hospital, Paris, neuropsychologist);

Eye movement: Sophie Rivaud-Pechoux, PhD (ICM, Paris, coordination); Elodie Hainque, MD, PhD (Pitié-Salpêtrière Hospital, Paris);

Sleep assessment: Isabelle Arnulf, MD, PhD (Pitié-Salpêtrière Hospital, Paris, coordination), Smaranda Leu Semenescu, MD (Pitié-Salpêtrière Hospital, Paris), Pauline Dodet, MD (Pitié-Salpêtrière Hospital, Paris);

Genetic data: Jean-Christophe Corvol, MD, PhD (Pitié-Salpêtrière Hospital, Paris, coordination), Graziella Mangone, MD, PhD (Pitié-Salpêtrière Hospital, Paris, co-coordination), Samir Bekadar, MS

(Pitié-Salpêtrière Hospital, Paris, biostatistician), Alexis Brice, MD (ICM, Pitié-Salpêtrière Hospital, Paris), Suzanne Lesage, PhD (INSERM, ICM, Paris, genetic analyses);

Metabolomics: Fanny Mochel, MD, PhD (Pitié-Salpêtrière Hospital, Paris, coordination), Farid Ichou, PhD (ICAN, Pitié-Salpêtrière Hospital, Paris), Vincent Perlbarg, PhD, Pierre and Marie Curie University), Benoit Colsch, PhD (CEA, Saclay), Arthur Tenenhaus, PhD (Supelec, Gif-sur-Yvette, data integration);

Brain MRI data: Stéphane Lehericy, MD, PhD (Pitié-Salpêtrière Hospital, Paris, coordination), Rahul Gaurav, MS, (Pitié-Salpêtrière Hospital, Paris, data analysis), Nadya Pyatigorskaya, MD, PhD, (Pitié-Salpêtrière Hospital, Paris, data analysis); Lydia Yahia-Cherif, PhD (ICM, Paris, Biostatistics), Romain Valabregue, PhD (ICM, Paris, data analysis), Cécile Galléa, PhD (ICM, Paris);

Datscan imaging data: Marie-Odile Habert, MCU-PH (Pitié-Salpêtrière Hospital, Paris, coordination);

Voice recording: Dijana Petrovska, PhD (Telecom Sud Paris, Evry, coordination), Laetitia Jeancolas, MS (Telecom Sud Paris, Evry);

Study management: Alizé Chalançon (Pitié-Salpêtrière Hospital, Paris, Project manager), Carole Dongmo-Kenfack (Pitié-Salpêtrière Hospital, Paris, clinical research assistant); Christelle Laganot (Pitié-Salpêtrière Hospital, Paris, clinical research assistant), Valentine Maheo (Pitié-Salpêtrière Hospital, Paris, clinical research assistant), Manon Gomes (Pitié-Salpêtrière Hospital, Paris, clinical research assistant)

Study sponsoring: The ICEBERG Study was funded by the Programme d'investissements d’avenir (ANR-10-IAIHU-06), the Paris Institute of Neurosciences – IHU (IAIHU-06), the Agence Nationale de la Recherche (ANR-11-INBS-0006), and Électricité de France (Fondation d’Entreprise EDF).

NCER-PD/LuxPARK consortium

We would like to thank all participants of the Luxembourg Parkinson’s Study for their important support to our research. Furthermore, we acknowledge the joint effort of the National Centre of Excellence in Research on Parkinson’s Disease (NCER-PD) Consortium members from the partner institutions Luxembourg Centre for Systems Biomedicine, Luxembourg Institute of Health, Centre Hospitalier de Luxembourg, and Laboratoire National de Santé generally contributing to the Luxembourg Parkinson’s Study as listed below:

Geeta ACHARYA[2], Gloria AGUAYO[2], Myriam ALEXANDRE[2], Muhammad ALI[1], Wim AMMERLANN[2], Giuseppe ARENA[1], Rudi BALLING[1], Michele BASSIS[1], Katy BEAUMONT[2], Regina BECKER[1], Camille BELLORA[2], Guy BERCHEM[3], Daniela BERG[11], Alexandre BISDORFF[5], Ibrahim BOUSSAAD[1], Kathrin BROCKMANN[11], Jessica CALMES[2], Lorieza CASTILLO[2], Gessica CONTESOTTO[2], Nico DIEDERICH[3], Rene DONDELINGER[5], Daniela ESTEVES[2], Guy FAGHERAZZI[2], Jean-Yves FERRAND[2], Manon GANTENBEIN[2], Thomas GASSER[11], Piotr GAWRON[1], Soumyabrata GHOSH[1], Marijus GIRAITIS[2],[3], Enrico GLAAB[1], Elisa GÓMEZ DE LOPE[1], Jérôme GRAAS[2], Mariella GRAZIANO[17], Valentin GROUES[1], Anne GRÜNEWALD[1], Wei GU[1], Gaël HAMMOT[2], Anne-Marie HANFF[2,20,21], Linda HANSEN[1,3], Michael HENEKA[1], Estelle HENRY[2], Sylvia HERBRINK[6], Sascha HERZINGER[1], Michael HEYMANN[2], Michele HU[8], Alexander HUNDT[2], Nadine JACOBY[18], Jacek JAROSLAW LEBIODA[1], Yohan JAROSZ[1], Sonja JÓNSDÓTTIR[2], Quentin KLOPFENSTEIN[1], Jochen KLUCKEN[1,2,3], Rejko KRÜGER[1,2,3], Pauline LAMBERT[2], Zied LANDOULSI[1], Roseline LENTZ[7], Inga LIEPELT[11], Robert LISZKA[14], Laura LONGHINO[3], Victoria LORENTZ[2], Paula Cristina LUPU[2], Tainá M. MARQUES[1], Clare MACKAY[10], Walter MAETZLER[15], Katrin MARCUS[13], Guilherme MARQUES[2], Patricia MARTINS CONDE[1], Patrick MAY[1], Deborah MCINTYRE[2], Chouaib MEDIOUNI[2], Francoise MEISCH[1], Myriam MENSTER[2], Maura MINELLI[2], Michel MITTELBRONN[1,4], Brit MOLLENHAUER[12], Friedrich MÜHLSCHLEGEL[4], Romain NATI[3], Ulf NEHRBASS[2], Sarah NICKELS[1], Beatrice NICOLAI[3], Jean-Paul NICOLAY[19], Fozia NOOR[2], Marek OSTASZEWSKI[1], Clarissa P. C. GOMES[1], Sinthuja PACHCHEK[1], Claire PAULY[1,3], Laure PAULY[2,20], Lukas PAVELKA[1,3], Magali PERQUIN[2], Nancy E. RAMIA[1,] Rosalina RAMOS LIMA[2], Armin RAUSCHENBERGER[1], Rajesh RAWAL[1], Dheeraj REDDY BOBBILI[1], Kirsten ROOMP[1], Eduardo ROSALES[2], Isabel ROSETY[1], Estelle SANDT[2], Stefano SAPIENZA[1], Venkata SATAGOPAM[1], Margaux SCHMITT[2], Sabine SCHMITZ[1], Reinhard SCHNEIDER[1], Jens SCHWAMBORN[1], Amir SHARIFY[2], Ekaterina SOBOLEVA[1], Kate SOKOLOWSKA[2], Hermann THIEN[2], Elodie THIRY[3], Rebecca TING JIIN LOO[1], Christophe TREFOIS[1], Johanna TROUET[2], Olena TSURKALENKO[2], Michel VAILLANT[2], Mesele VALENTI[2], Gilles VAN CUTSEM[1,3], Carlos VEGA[1], Liliana VILAS BOAS[3], Maharshi VYAS[1], Richard WADE-MARTINS[9], Paul WILMES[1], Evi WOLLSCHEID-LENGELING[1], Gelani ZELIMKHANOV[3]

1. Luxembourg Centre for Systems Biomedicine, University of Luxembourg, Esch-sur-Alzette, Luxembourg

2. Luxembourg Institute of Health, Strassen, Luxembourg
3. Centre Hospitalier de Luxembourg, Strassen, Luxembourg
4. Laboratoire National de Santé, Dudelange, Luxembourg
5. Centre Hospitalier Emile Mayrisch, Esch-sur-Alzette, Luxembourg
6. Centre Hospitalier du Nord, Ettelbrück, Luxembourg
7. Parkinson Luxembourg Association, Leudelange, Luxembourg
8. Oxford Parkinson's Disease Centre, Nuffield Department of Clinical Neurosciences, University of Oxford, Oxford, UK
9. Oxford Parkinson's Disease Centre, Department of Physiology, Anatomy and Genetics, University of Oxford, South Parks Road, Oxford, UK
10. Oxford Centre for Human Brain Activity, Wellcome Centre for Integrative Neuroimaging, Department of Psychiatry, University of Oxford, Oxford, UK
11. Center of Neurology and Hertie Institute for Clinical Brain Research, Department of Neurodegenerative Diseases, University Hospital Tübingen, Germany
12. Paracelsus-Elena-Klinik, Kassel, Germany
13. Ruhr-University of Bochum, Bochum, Germany
14. Westpfalz-Klinikum GmbH, Kaiserslautern, Germany
15. Department of Neurology, University Medical Center Schleswig-Holstein, Kiel, Germany
16. Department of Neurology Philipps, University Marburg, Marburg, Germany
17. Association of Physiotherapists in Parkinson's Disease Europe, Esch-sur-Alzette, Luxembourg
18. Private practice, Ettelbruck, Luxembourg
19. Private practice, Luxembourg, Luxembourg
20. Faculty of Science, Technology and Medicine, University of Luxembourg, Esch-sur-Alzette, Luxembourg
21. Department of Epidemiology, CAPHRI School for Public Health and Primary Care, Maastricht University Medical Centre+, Maastricht, the Netherlands
